# No Evidence for an Awareness-Dependent Emotional Modulation of the Attentional Blink

**DOI:** 10.3389/fpsyg.2019.02422

**Published:** 2019-10-25

**Authors:** Jelena Galojan, Cornelia Kranczioch

**Affiliations:** Neurocognition and Functional Neurorehabilitation Group, Neuropsychology Lab, Department of Psychology, University of Oldenburg, Oldenburg, Germany

**Keywords:** awareness-dependent modulation of the AB, emotional modulation of the AB, attentional blink, emotional attentional blink, training effect of the attentional blink

## Abstract

Pictures of faces with emotional expressions presented before a temporal attention task have been reported to affect temporal attention in an awareness-dependent manner: Awareness of a fearful face was linked to an increased deficit in the temporal attention task, while preventing the face from reaching awareness was linked to a decreased deficit, both relative to neutral faces. Here we report the results of two temporal attention experiments which aimed to extend and conceptually replicate this basic finding. The temporal attention task was preceded by an unmasked or a masked fearful face on a trial-by-trial basis. In both experiments the finding of an awareness-dependent emotional modulation of temporal attention through fearful faces could not be replicated, even when data were pooled across experiments. Pooling of experiments indicated however that, independent of awareness level, fearful faces can be associated with slightly worse temporal attention performance than neutral faces, and suggested a lag-specific practice effect in terms of a reduced deficit in temporal attention in the second half of the experiment.

## Introduction

A paradigm popular in the study of the temporal limits of attention is the attentional blink (AB). Here, sequences of stimuli are presented in rapid serial visual presentation (RSVP), at a rate of about 10 per second. Sequences contain two target stimuli at varying positions that have to be identified or detected, denoted T1 and T2. The detection or identification of T2 is impaired if it appears about 200 – 500 ms after T1 (Raymond et al., 1992; Shapiro et al., 1994; Chun and Potter, 1995; Meng and Potter, 2011). Pictures of fearful faces preceding the RSVP have been reported to negatively affect temporal attention compared to disgust faces (Vermeulen et al., 2009). For RSVP-preceding emotional stimulus material other than faces a beneficial effect of positive pictures compared to negative pictures was reported for the AB, while the same study did not find an effect of negative compared to neutral pictures (Olivers and Nieuwenhuis, 2006). One study investigated whether this emotional modulation of the AB is awareness-dependent (Qian et al., 2012). In this study, compared to neutral faces, masked fearful faces preceding the RSVP sequence significantly decreased the AB magnitude, whereas unmasked fearful faces significantly increased the AB magnitude. The opposite effects were suggested to be caused by different neural mechanisms underlying conscious and unconscious emotional processing. In particular, the effect of masked fearful faces was proposed to result from them attracting more attention than unmasked fearful faces as a consequence of being processed by a fast, subcortical pathway. This processing advantage would lead to a diffuse attentional state reducing the AB effect (Qian et al., 2012). Though the idea of a fast subcortical route for the processing of emotional stimuli in the human brain has been challenged (Pessoa and Adolphs, 2010), the origin of the opposite impact of masked, and unmasked emotional faces on the AB remains unknown. Investigating it would require for instance functional magnetic resonance (fMRI) or event-related potential (ERP) approaches. For these approaches some major adjustments to the experimental design are however needed. A necessary adjustment is the reduction of conditions to allow for a sufficient number of repetitions of each condition for analysis. The original study by Qian et al. (2012) used eight T1-T2 lags, while neurophysiological and neuroimaging studies use up to three (e.g., Vogel et al., 1998; Kranczioch et al., 2003, 2005; Marois et al., 2004). A desirable adjustment is to move from a between- to a within-subjects design if the experimental manipulation of interest does not require a between-subjects design. Though the original study by Qian et al. (2012) used a between-subjects design, their results suggest that this is no precondition for the effect of emotional faces on the AB. That is, trials with emotional faces were clearly different from intermixed trials with neutral faces, arguing for a short-lasting phasic effect of the emotional face. In support, across both experiments performance for neutral faces was comparable (Qian et al., 2012). Within-subject designs have the general advantage that fewer participants are needed and that there is less variance due to differences between participants, both being of particular relevance when data collection becomes time and/or cost intensive or when differences between conditions might be small. Aim of this study was to implement these adjustments and to test whether the basic findings of Qian et al. (2012) could be conceptually replicated in a purely behavioral study. If successful, the present study would prepare the way for subsequent neuroimaging or neurophysiological studies investigating the neural correlates of the emotional modulation of the AB.

## Methods and Results

### Experiment 1

The first experiment tested the influence of a masked or unmasked emotional face on temporal attention in an AB setup with only two lags for T2. Each participant performed a masked and an unmasked condition, with neutral trials intermixed. Additional minor changes compared to the original study by Qian et al. (2012) were implemented as well. First, the RSVP stream consisted of capital letters and targets were digits, while in the Qian et al. (2012) study the reverse was the case. We opted for digits as targets with an environment with restricted response options in mind, such as the MRI scanner. That is, in comparison to a lab environment where a standard keyboard can be used to collect a large number of different responses without the need to memorize button-response associations, response options are much more limited in an MRI scanner and button-response options need to be memorized. We reasoned that with digits as targets the paradigm could be more easily translated to such conditions. Second, we used the recent state-of-the-art Radboud face database (Langner et al., 2010) of Caucasian faces as we deemed this database suitable for our participants, Qian et al. (2012) used the Japanese and Caucasian Facial Expressions of Emotion (JACFEE) and Neutral Faces (JACNeuF) set by Matsumoto and Ekman (1988). Third, presentation durations of all stimuli were adjusted to comply with our combination of hardware and software (see below). All differences between the study by Qian et al. (2012) and the present study are listed again in Table 1.

**TABLE 1:** Differences between Qian et al. (2012) and present study.

	**Qian et al., 2012**	**Present study**	**Reason for change**
T2 lags	Eight (1–8)	Two (2,7)	Reduction of experimental conditions
Design emotion manipulation	Between-subjects	Within-subjects	Reduction of variance
Face database	Japanese and Caucasian facial expressions of emotion and neutral faces (Matsumoto and Ekman, 1988^∗^)	Radboud face database (Oliver et al., 2010)	Radboud face database is more recent and faces match the social group of the study population
Social group face stimuli	Caucasian and Japanese in unknown proportion	Caucasian	Stimuli were taken from a recent state-of-the-art database (see above); no *a priori* reason to expect an influence of the social group of the face stimuli
Social group participants	Chinese (probably)	German	Convenience sample; no *a priori* reason to expect an influence of social group of participants
Face presentation	Color and full head	Grayscale, masked by an oval to highlight emotional features	To highlight emotional features and to attenuate non-emotional features (e.g., hair and unclean skin)
Distracters Targets	Digits Capital letters (Latin letters)	Experiment. 1: Capital letters Experiment 1: Digits	Digits as targets are better compatible with the restricted response options of the MRI-environment
		Experiment 2: Digits Experiment 2: Capital letters	For replicating the findings of Experiment 1 distracters and targets were swapped to match (Qian et al., 2012)
RSVP color	Black on gray	White on black	Better visibility in dark environment (piloting)
Trial start	Self-paced	Automatic	To achieve fixed trial and experiment duration as desirable for an fMRI protocol
Fixation cross	Green	Red	Better visibility in dark environment (piloting)
Fixation cross duration	500 ms	1000 ms	To allow for preparation for next trial as trials started automatic rather than self-paced
Face stimulus pair presentation durations	30 ms/50 ms	33 ms/50 ms	To match face presentation durations with screen refresh rate
Blank between second face and onset of RSVP	20 ms	50 ms	20 ms SOA associated with increased T1 misses (piloting)
RSVP stimuli presentation duration	90 ms	66 ms	Comparability with own previous work and the majority of AB studies where an SOA of 100 ms is used with a non-zero ISI
SOA/ISI	90 ms/0 ms	100 ms/33 ms	

*^∗^As cited by Qian et al. (2012): Matsumoto and Ekman (1988). Japanese and Caucasian Facial Expressions of Emotion (JACFEE) and Neutral Faces (JACNeuF). Report available from Intercultural and Emotion Research Laboratory, Department of Psychology, San Francisco State University.*

#### Subjects

Twenty subjects participated in exchange for 8€ reimbursement (10 females, 10 males, and age: *M* = 23.15 years, *SD* = 3.59 years). Sample size was determined with G^∗^Power 3.1 (Faul et al., 2007) as described in the Supplementary Data Sheet S1. All participants had normal or corrected-to-normal visual acuity and were free of past or current psychiatric or neurological illness. All participants provided written informed consent prior to the experiment. The study was approved by the ethical review board of the University of Oldenburg.

#### Stimuli

**FIGURE 1:**
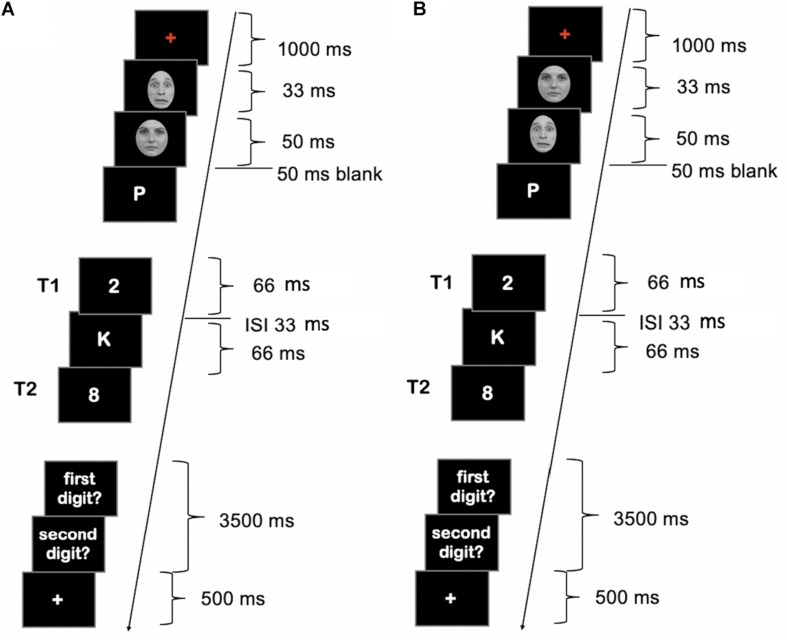
Schematic illustration of the attentional blink (AB) stream in Experiment 1. **(A)** Masked condition. **(B)** Unmasked condition. Face images were obtained from the Radboud Faces Database (Langner et al., 2010) and are reprinted in line with the guidelines of the database.

The emotional faces were black and white photographs, which were selected from the Radboud Faces Data Base (Oliver et al., 2010). Faces were presented in black-and white and overlaid with an ellipse to hide hair, accessories and clothes, in order to highlight the faces. They included eight fearful and 24 neutral Caucasian faces. Each category of faces was balanced by gender. In each trial, faces were drawn randomly with replacement from their respective category set. The RSVP stream consisted of 21 items, including T1 and T2. Distractors were all letters except E, F, I, O, Q, U, X, and Y. Targets were all digits from 1 to 8. All RSVP stimuli were shown in white on a black background (see Figure 1). The dimensions of the pictures were 681 × 1024 pixels. All stimuli were shown on a gray background. Letter size was 200 points.

#### Procedures

The subjects were tested individually in a dimly lit and soundproofed room. The AB stream was shown on a Dell PC (Intel^®^ Core^TM^ i7 2.93 GHz/8 GB RAM) with a screen refresh rate of 120 Hz and a monitor resolution of 1920 × 1080. The distance between the screen and the participants’ eyes was approximately 125 cm. The program Presentation version 19.9 (Neurobehavioral Systems- Berkeley, CA, United States) was used for experimental control. Each trial began with a 1000 ms presentation of a red fixation cross. This was followed by the presentation of two face photographs. The first face (neutral or fearful) appeared for 33 ms and was immediately backward masked with another face (neutral or fearful) lasting 50 ms. In the neutral-neutral condition both faces were neutral, in the fearful-neutral condition the fearful face was shown first and then masked by the neutral face, and in the neutral-fearful condition the fearful face came second and was therefore unmasked. Accuracy of the face presentation durations was confirmed after the experiment for each participant. Logfiles indicated that possibly the presentation duration of the faces was unstable for up to five trials of the first ten trials of the main experiment. That is, according to the logfiles, in a small fraction of trials faces might have been presented slightly longer or shorter than intended. On average, four trials of 216 were affected (*M* = 4.1, *SD* = 0.81, corresponding to *M* = 1.9%/*SD* = 0.36 of trials). Masking conditions (masked and unmasked) were blocked, and it was balanced across participants which masking condition would run first. Therefore, both masking conditions were affected similarly by the possible instability in face presentation duration. However, a longer presentation duration of the first face would in particular affect the visibility of fearful faces in the masked condition and might result in mimicking the effect expected for unmasked fearful faces, that is, a decrease in AB performance. To check for this possibility, for the masked condition performance was compared in the 19 trials with a reported instability in first face duration (range 35–52 ms instead of 33 ms) and the 12 trials with a reported instability in second face duration (range 48–55 ms instead of 50 ms). Of the 19 trials with reported first face instability, 15 trials were valid (i.e., correct T1 response). Of those, ten trials were associated with a correct T2 response and five with an incorrect T2 response, corresponding to a hit rate of 66%. Of the 12 trials with reported second face instability, ten trials were valid. Of those, five trials were associated with a correct T2 response and five with an incorrect T2 response, corresponding to a hit rate of 50%. That is, there was no indication that the trials with reported timing instability for the first face would increase the AB. Therefore, all valid trials were included in the analysis.

A blank screen was shown for 50 ms between the second face and the start of the RSVP stream. The RSVP stream consisted of 21 letters (see Figure 1) including the targets. Distracter RSVP elements were drawn randomly from the set of distracters letters. The position of T1 in the stream varied between position 7–12 of the RSVP sequence. T2 appeared either as the second or seventh stimulus after the T1 (lag 2 or lag 7, respectively). Each RSVP stimulus was presented for 66 ms with an interstimulus interval (ISI) of 33 ms (see Figure 1). The RSVP stream was followed by a 500 ms blank screen before the response screens appeared. Responses were possible for up to 3500 ms. Participants were instructed to type in the digits after each trial in the order they saw them in the AB stream. Participants were encouraged to guess if they were not sure. Responses were counted as correct regardless of the order in which they were given. If responses were faster than 3500 ms, a white fixation cross appeared for the remaining response period. A trial ended with the (continuing) presentation of a white fixation cross, whose duration varied randomly between 100 and 500 ms in 100 ms steps. T1 and T2 tasks were chosen to allow for easy transfer of the setup to an fMRI setup with limited response options.

**FIGURE 2:**
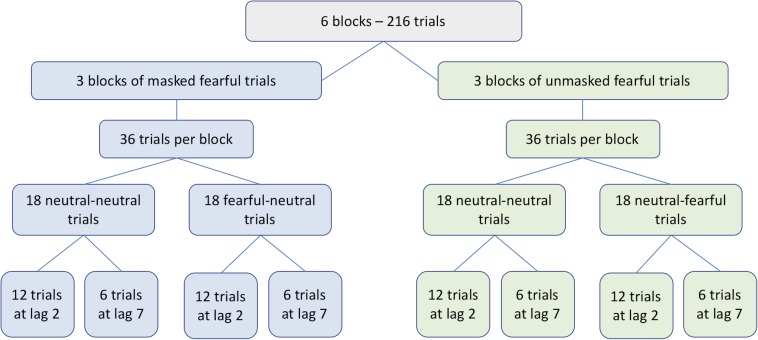
Schematic illustration of the experimental design in all experiments.

The experiment consisted of 216 trials across six blocks – 3 blocks with unmasked fearful faces and 3 blocks with masked fearful faces. That is, in contrast to the original publication (Qian et al., 2012), a within-design rather than a between-design was chosen. There were breaks of 30 s between the blocks and participants could start each new block by pressing any button on the response keyboard. As shown in Figure 2 each block consisted of 36 randomly intermixed trials – 18 were neutral-neutral trials and the other 18 were either fearful-neutral or neutral-fearful trials. For each condition 12 trials showed T2 at lag 2 and 6 trials showed T2 at lag 7. The number of trials in the experiment was maximized for T2 lag 2 as would be in a neurophysiological or neuroimaging study focusing on T2 processing during the critical AB period. This is in contrast to the original publication (Qian et al., 2012) where all T2 lags between lag 1 and lag 8 were tested. Lag 7 targets were included to demonstrate the presence of an AB and to be able to detect any lag-specific effects of emotional faces and masking, if present. The three blocks per condition were run in blocked order, with starting condition counterbalanced across participants. Two practice trials with reduced speed and four practice trials with normal speed were conducted before the experiment. Practice trials contained examples of both the masked and the unmasked condition.

#### Statistical Analyses

Performance in the AB experiment was calculated as the percentage of correct T2 responses in all trials with correct T1 responses (T2|T1). Percentages were submitted to a repeated measures 2 × 2 × 2 × 2 ANOVA with within-group factors emotional condition (fearful, neutral), masking condition (masked, unmasked) and T2 lag (2,7) and between-group factor group (unmasked condition first and masked condition first). Note that in this ANOVA model, neutral/neutral trials of the blocks with masked fearful faces and neutral/neutral trials of the blocks with unmasked fearful faces are treated as separate conditions. This was done to account for possible time-on-task effects and for any tonic effects masking or not masking the emotional face might exert on neutral/neutral trials in the corresponding blocks, and for better comparability of the results with Qian et al. (2012).

#### Results

**FIGURE 3:**
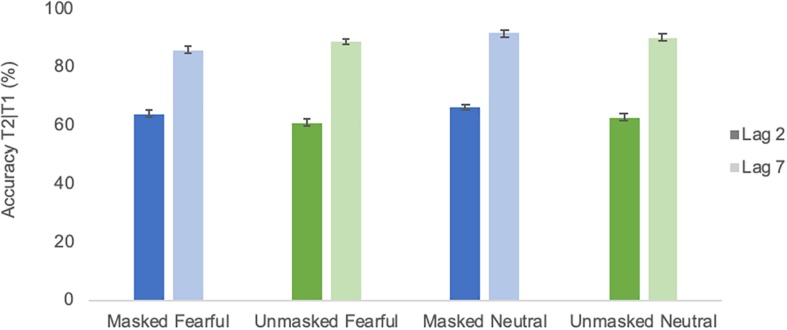
Average identification accuracy of T2 in different emotional conditions of Experiment 1. Error bars represent one standard error of the mean.

**FIGURE 4:**
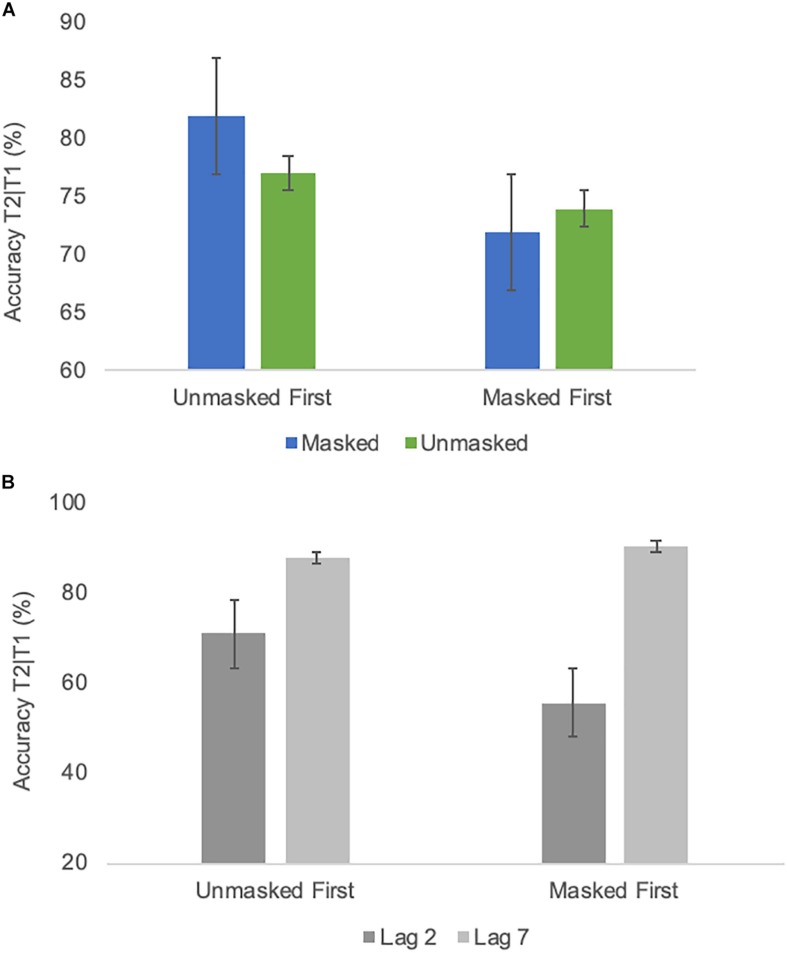
**(A)** Average identification accuracy of T2 in different masking conditions of Experiment 1 in a group comparison (starting with the unmasked condition/starting with the masked condition). **(B)** Average identification accuracy of T2 at different lags of Experiment 1 in a group comparison (starting with the unmasked condition/starting with the masked condition). Error bars represent one standard error of the mean.

As evident from Figure 3, an AB-like pattern was evident across masking and emotion conditions. All ANOVA results are listed in Table 2. The ANOVA indicated a significant effect [*F*(1,18) = 9.13; *p* < 0.01; $\eta_{\text{p}}^{2}$ = 0.34] of emotional condition. Trials with only neutral faces before the RSVP stream were associated with a slightly better detection accuracy than trials with masked or unmasked emotional faces (*M* = 77.6% and SE = 2.4 vs. *M* = 74.8% and SE = 2.6). The T2 lag effect was again highly significant [*F*(1,18) = 51.65; *p* ≤ 0.0001; $\eta_{\text{p}}^{2}$ = 0.74], confirming the AB. There was a significant interaction between masking condition and group [*F*(1,18) = 8.19; *p* = 0.01; $\eta_{\text{p}}^{2}$ = 0.31] due to worse performance in the condition with which a group started (see Figure 4A). Additionally, there was also a significant interaction between group and T2 lag [*F*(1,18) = 6.35; *p* = 0.02; $\eta_{\text{p}}^{2}$ = 0.26], reflecting that the group that started with the masked condition performed worse at lag 2 than the other group (group masked first *M* = 55.7% and SE = 5.3, group unmasked first *M* = 71% and SE = 5.3), while performance was comparable for lag 7 (group masked first *M* = 90.3% and SE = 2.9, group unmasked first *M* = 87,7% and SE = 2.9) (see Figure 4B). None of the effects involving an interaction of the factors masking condition and emotional condition were significant (cf. Table 2). To test the confidence in the latter, an analysis of effects across matched models of a Bayesian repeated measures 2 × 2 × 2 × 2 ANOVA (Jasp Team, 2019, Version 0.10.2; Rouder et al., 2012) with factors emotional condition, masking condition, T2 lag, and group was run. This analysis yielded the Bayes Inclusion factor based on matched models (BF_*incl*_, also called Baws factor) that represents the evidence for all models containing a particular effect compared to equivalent models stripped of that effect. Results indicated that evidence for H0 is anecdotal to moderate, with all BF_*incl*_ smaller than 0.279 with the exception of the four-way interaction with a BF_*incl*_ = 0.349. That is, there is anecdotal to moderate evidence that models including the interaction masking condition × emotion condition are *not* better than models without the interaction masking condition × emotional condition. The complete results of the Bayesian repeated measures ANOVA are provided as Supplementary Table S1.

**TABLE 2:** ANOVA results Experiment 1.

	***df***	***F***	***p***	**$\eta_{\text{p}}^{2}$**
Masking	1,18	1.08	0.31	0.06
Emotion	1,18	**9**.**13**	**<0.01**	0.34
Lag	1,18	**51.65**	**<0.0001**	0.74
Group	1,18	1.72	0.21	0.09
Masking × Group	1,18	**8.19**	**0.010**	0.31
Emotion × Group	1,18	0.60	0.45	0.03
Lag × Group	1,18	**6.35**	**0.02**	0.26
Masking × Emotion	1,18	0.93	0.35	0.05
Masking × Lag	1,18	3.33	0.09	0.16
Emotion × Lag	1,18	0.28	0.60	0.02
Masking × Emotion × Group	1,18	0.12	0.74	0.01
Masking × Lag × Group	1,18	3.85	0.07	0.18
Emotion × Lag × Group	1,18	0.03	0.87	<0.01
Masking × Emotion × Lag	1,18	0.68	0.42	0.04
Masking × Emotion × Lag × Group	1,18	0.63	0.44	0.03

*In bold are highlighted the *F* and *p* values of statistically significant effects.*

### Experiment 2

Experiment 1 revealed a significant effect of emotion with neutral faces associated with slightly better performance than emotional faces. In contrast to the original study (Qian et al., 2012) where impaired performance was associated with unmasked emotional faces only, this effect was not modulated by masking condition. Moreover, the group starting with the unmasked condition displayed a larger deficit at lag 2 across all conditions. The second experiment tested the reliability of these findings. The experimental setup was slightly adapted in that now letters were used as targets and digits as distracters, similar to the original study by Qian et al. (2012).

#### Subjects

Twenty new subjects participated in exchange for 8€ reimbursement (10 females, 10 males, and age: *M* = 25.25 years, *SD* = 3.61 years). All participants had normal or corrected-to-normal visual acuity and were free of past or current psychiatric or neurological illness. All participants provided written informed consent prior to the experiment. The study was approved by the ethical review board of the University of Oldenburg.

#### Stimuli

**FIGURE 5:**
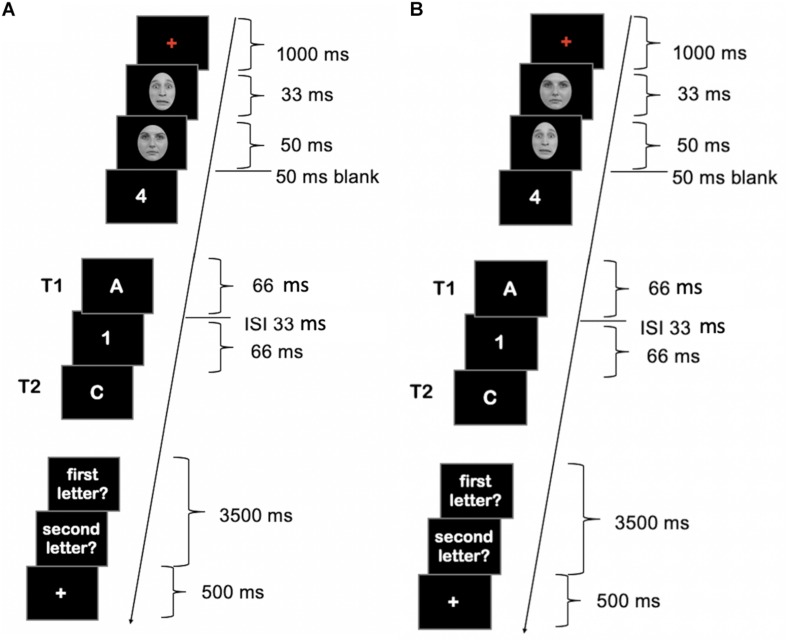
Schematic illustration of the AB stream in Experiment 2. **(A)** Masked condition. **(B)** Unmasked condition. Face images were obtained from the Radboud Faces Database (Langner et al., 2010) and are reprinted in line with the guidelines of the database.

Stimulus parameters were identical to Experiment 1 with the exception that in Experiment 2 distractors were digits from 0 to 9 and targets were the letters A, B, C, D, G, H, K, and L (see Figure 5).

#### Procedures

**FIGURE 6:**
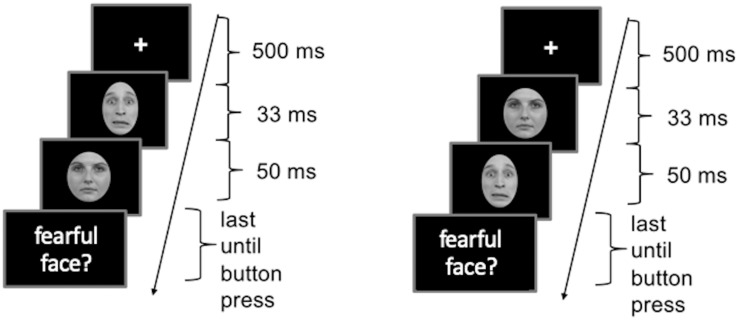
Schematic illustration of the procedures in the forced choice experiment in Study 2. **(A)** Masked condition. **(B)** Unmasked condition. Face images were obtained from the Radboud Faces Database (Langner et al., 2010) and are reprinted in line with the guidelines of the database.

Procedures of Experiment 2 were the same as in Experiment 1 with the exception that Experiment 2 was run using Presentation version 20.1. In addition, to confirm the efficiency of the masking parameters, after the AB experiment a forced choice experiment was conducted. The same stimuli of Caucasian faces as used for the main experiment were used (see Figure 6). The forced-choice experiment contained two blocks – one for the masked condition and one for the unmasked condition – consisting of 60 emotional face trials, respectively. Both blocks also had a randomly intermixed neutral condition with 30 trials. Each trial began with a fixation cross for 500 ms followed by two pictures of emotional or neutral faces shown for 33 and 50 ms, respectively, in the style of the AB experiment. Face pairs were either neutral-neutral, fearful-neutral (masked condition) or neutral-fearful (unmasked condition). After the second face participants had to decide whether they saw a fearful face by pressing either *yes* or *no* on the keyboard. The response time was not restricted (see Figure 6). In the blocked design all participants started with the masked condition.

#### Statistical Analyses

Performance in the AB experiment was calculated as the percentage of correct T2 responses in all trials with correct T1 responses (T2|T1). Percentages were submitted to a repeated measures 2 × 2 × 2 × 2 ANOVA with within-group factors emotional condition (fearful, neutral), masking condition (masked, unmasked) and T2 lag (2,7) and between-group factor group (unmasked condition first and masked condition first). Neutral/neutral trials of the blocks with masked fearful faces and neutral/neutral trials of the blocks with unmasked fearful faces were again treated as separate conditions. For the forced choice task, hit rates, false alarm rates and d’ (Macmillan, 1986) were calculated separately for each block.

#### Results

##### Attentional blink

**FIGURE 7:**
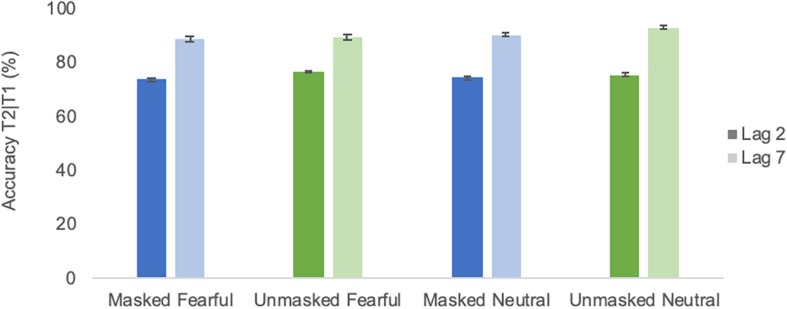
Average identification accuracy of T2 in different emotional conditions of Experiment 2. Error bars represent one standard error of the mean.

**FIGURE 8:**
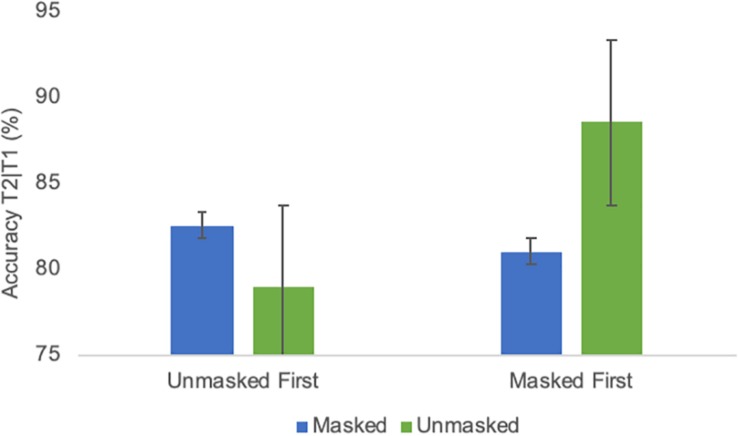
Average identification accuracy of T2 in different masking conditions of Experiment 2 in a group comparison (starting with the unmasked condition/starting with the masked condition). Error bars represent one standard error of the mean.

As depicted in Figure 7, performance was very comparable to Experiment 1. All ANOVA results are listed in Table 3. The emotion effect observed in Experiment 1 was not significant [*F*(1,18) = 0.72; *p* = 0.407; $\eta_{\text{p}}^{2}$ = 0.5]. The lag effect was significant [*F*(1,18) = 31.6; *p* ≤ 0.0001; $\eta_{\text{p}}^{2}$ = 0.64], confirming the AB. Moreover, there was a significant interaction between masking and group [*F*(1,18) = 17.7; *p* = 0.001; $\eta_{\text{p}}^{2}$ = 0.5] as again, performance was overall worse in the condition with which a group began the experiment (see Figure 8 None of the effects involving an interaction of the factors masking and emotion were significant (cf. Table 3). The analysis of effects across matched models of a Bayesian repeated measures 2 × 2 × 2 × 2 ANOVA with factors emotional condition, masking condition, T2 lag and group indicated that evidence for H0 is anecdotal to moderate, with BF_*incl*_ ranging from 0.238 for the interaction emotional condition × masking condition to 0.511 for the interaction masking condition × emotional condition × group. That is, as for Experiment 1, there is anecdotal to moderate evidence that models including the interaction masking condition × emotion condition are *not* better than models without the interaction masking condition × emotional condition. The complete results of the Bayesian repeated measures ANOVA are provided as Supplementary Table S2.

**TABLE 3:** ANOVA results Experiment 2.

	***df***	***F***	***p***	**$\eta_{\text{p}}^{2}$**
Masking	1,18	2.02	0.17	0.10
Emotion	1,18	0.72	0.41	0.04
Lag	1,18	**31.60**	**0.0001**	0.64
Group	1,18	0.64	0.43	0.03
Masking × Group	1,18	**17.66**	**0.001**	0.50
Emotion × Group	1,18	< 0.01	0.99	< 0.0001
Lag × Group	1,18	0.02	0.88	< 0.01
Masking × Emotion	1,18	< 0.001	0.99	< 0.0001
Masking × Lag	1,18	< 0.01	0.96	< 0.001
Emotion × Lag	1,18	1.00	0.33	0.05
Masking × Emotion × Group	1,18	2.21	0.16	0.11
Masking × Lag × Group	1,18	3.88	0.06	0.18
Emotion × Lag × Group	1,18	0.20	0.66	0.01
Masking × Emotion × Lag	1,18	0.71	0.41	0.04
Masking × Emotion × Lag × Group	1,18	0.02	0.90	< 0.01

*In bold are highlighted the *F* and *p* values of statistically significant effects.*

##### Forced choice task

**FIGURE 9:**
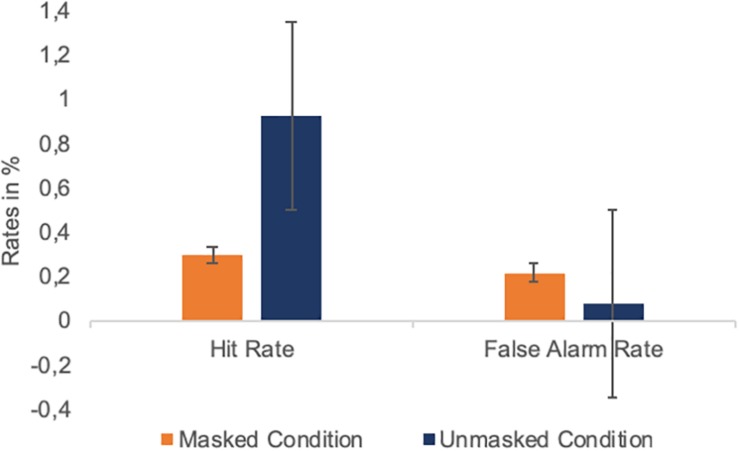
Average hit and false alarm rates in the forced choice experiment in Study 2. Error bars represent one standard error of the mean.

The results of the forced choice task are summarized in Figure 9. Hit rate was high for emotional faces in the unmasked condition (*M* = 0.92, *SD* = 0.09), while trials with only neutral faces presented intermixed with the unmasked emotional faces gave rise to only a small number of FAs (*M* = 0.1, *SD* = 0.1). FA rate was higher for the masked condition (*M* = 0.23, *SD* = 0.17) and close to the masked condition hit rate (*M* = 0.31, *SD* = 0.17). d′ was high for the unmasked condition (*d*′ = 3.04, SE = 0.73) and low for the masked condition (*d*′ = 0.28, SE = 0.4). This pattern confirms that the masking parameters used in the AB were efficient in modulating awareness of emotional faces.

### Combined Analysis Experiments 1 and 2

The study by Qian et al. (2012) tested 21 participants in each experiment. Participant number in the present study was 20 participants for both experiments. In order to achieve higher power for detecting an awareness-dependent effect of the emotional faces if present, data from both experiments were pooled.

Data were analyzed with an ANOVA with factors masking (masked, unmasked), emotion (neutral, fearful), lag (2,7), and group (unmasked condition first, masked condition first). Combined sample size was *n* = 40. All results of the main ANOVA are listed in Table 4. ANOVA results indicated a significant effect of emotion [*F*(1,38) = 6.2; *p* = 0.017; $\eta_{\text{p}}^{2}$ = 0.14]. The emotion effect was due to a slightly better performance in the neutral compared to emotional face conditions [fearful *M* = 78.5% (SE = 2) vs. neutral *M* = 80.5% (SE = 1.8)]. A highly significant lag effect was also evident [*F*(1,83) = 69.5; *p* < 0.0001; $\eta_{\text{p}}^{2}$ = 0.39] as were an interaction of factors group and masking [*F*(1,38) = 23.77; *p* = 0.016; $\eta_{\text{p}}^{2}$ = 0.31] and of factors group, masking and lag [*F*(1,32) = 7.44; *p* = 0.01; $\eta_{\text{p}}^{2}$ = 0.16]. None of the effects involving an interaction of the factors masking and emotion were significant (cf. Table 4). Regarding latter, an analysis of effects across matched models of a Bayesian repeated measures 2 × 2 × 2 × 2 ANOVA with factors emotional condition, masking condition, T2 lag and group indicated that evidence for H0 is moderate to anecdotal, with BF_*incl*_ ranging from 0.191 for the interaction emotional condition × masking condition to 0.297 for the four-way interaction. That is, also for the combined data of Experiments 1 and 2, there is anecdotal to moderate evidence that models including the interaction masking condition × emotion condition are *not* better than models without the interaction masking condition × emotional condition. The complete results of the Bayesian repeated measures ANOVA are provided as Supplementary Table S3.

**TABLE 4:** ANOVA results joint analysis Experiments 1 and 2.

	***df***	***F***	***p***	**$\eta_{\text{p}}^{2}$**
Masking	1,38	0.09	0.77	< 0.01
Emotion	1,38	**6.20**	**0.02**	0.14
Lag	1,38	**69.47**	**<0.0001**	0.65
Group	1,38	0.08	0.78	< 0.01
Masking × Group	1,38	**23.77**	**<0.0001**	0.39
Emotion × Group	1,38	0.20	0.66	< 0.01
Lag × Group	1,38	3.02	0.09	0.07
Masking × Emotion	1,38	0.42	0.52	0.01
Masking × Lag	1,38	0.84	0.37	0.02
Emotion × Lag	1,38	1.26	0.27	0.03
Masking × Emotion × Group	1,38	0.70	0.41	0.02
Masking × Lag × Group	1,38	**7.44**	**0.01**	0.16
Emotion × Lag × Group	1,38	0.04	0.84	< 0.01
Masking × Emotion × Lag	1,38	<0.01	0.98	< 0.0001
Masking × Emotion × Lag × Group	1,38	0.46	0.50	0.01

*In bold are highlighted the *F* and *p* values of statistically significant effects.*

The three-way interaction of the main ANOVA was followed by two 2 × 2 ANOVAS with factors group and masking. For the lag 2 ANOVA, the interaction group × masking was significant [*F*(1,38) = 25.8; *p* < 0.0001; $\eta_{\text{p}}^{2}$ = 0.41], while for the lag 7 ANOVA the interaction did not reach significance [*F*(1,38) = 1.4; *p* = 0.25; $\eta_{\text{p}}^{2}$ = 0.034]. The group × masking interaction for lag 2 was followed by simple comparisons, demonstrating that lag 2 performance increased by about 7% in the second half of the experiment irrespective of whether a group started with the masked [*t*(19) = −3.4; *p* = 0.003; masked (1st half) *M* = 63.2% (*SD* = 22.8) vs. unmasked (2nd half) 69.9% (*SD* = 21.8)] or the unmasked condition [*t*(19) = 3.75, *p* = 0.001; unmasked (1st half) *M* = 67.9% (*SD* = 11.9) vs. masked (2nd half) *M* = 75.9% (*SD* = 13.4)]. Performance did not differ significantly between masked and unmasked conditions when compared between groups within the first [*t*(38) = 0.81; *p* = 0.42] and within the second [*t*(38) = 1.1, *p* = 0.3] half of the experiment. Thus, a practice effect was evident that was restricted to lag 2 irrespective of masking condition.

To check for the possibility that the lag 2 practice effect covered an interaction between emotion and masking, we run an additional exploratory analysis in which lag 2 performance in the first half of the experiment was compared between groups, that is, the factor masking, so far a within-factor, became a between factor. An ANOVA was run with between-factor masking (masked and unmasked) and within-factor emotion (neutral and fearful). Again, there was no evidence for an interaction between masking and emotion [*F*(1,38) = 0.14, *p* = 0.71, $\eta_{\text{p}}^{2}$ = 0.004] or a masking main effect [*F*(1,38) = 0.66, *p* = 0.42, $\eta_{\text{p}}^{2}$ = 0.017]. The emotion effect did not reach significance [*F*(1,38) = 1.47, *p* = 0.23, $\eta_{\text{p}}^{2}$ = 0.037], likely because only half of the data entered this analysis.

In summary, as for the original analyses, the joined analysis of Experiments 1 and 2 did not reveal any evidence for an awareness-dependent modulation of the AB through fearful faces, though in general, fearful faces were associated with a slightly reduced hit rate compared to neutral faces. The joined analysis also indicated a practice effect that was restricted to lag 2.

## Discussion

Pictures of faces with emotional expressions have been found to affect temporal attention (Vermeulen et al., 2009), and the direction of the modulation by emotion has been reported to be awareness-dependent (Qian et al., 2012). The present study aimed to conceptually replicate and extend the finding that masked fearful faces preceding the AB stream decrease the AB whereas unmasked fearful faces increase the AB, both relative to neutral faces (Qian et al., 2012). The main results of two experiments can be summarized as follows: (1) A clear AB effect is evident in both experiments, i.e., detection or identification of T2 at lag 2 is more impaired than at lag 7, but this impairment is not modulated by emotional faces in an awareness-dependent manner. (2) Emotional faces preceding the RSVP stream slightly reduced T2 performance, irrespective of whether the fearful face is masked or unmasked and irrespective of T2 lag. (3) A time-on-task effect is evident for T2 performance. When data are collapsed across experiments it becomes evident that the time-on-task effect is specific for lag 2. The main results will be discussed in the following.

### No Evidence for an Awareness-Dependent Modulation of Temporal Attention

The failure of finding an awareness-dependent modulation of temporal attention as measured with the AB by images of fearful faces is in obvious contrast to the findings of Qian et al. (2012). We aimed for a conceptual and not a direct replication, therefore the two studies differed in several aspects. A major difference is that we incorporated a within-subjects design, because this should increase the probability of detecting an awareness-dependent modulation of temporal attention. That we did not observe such modulation could mean that processing masked and unmasked emotional stimuli within a single session abolishes their respective effects on the AB. The results of a *post hoc* analysis that included only the first half of a session, and therefore between subjects either masked or unmasked emotional stimuli, did however similarly not provide evidence for an awareness-dependent effect of masking. Thus, it is very unlikely that the within-subjects design is the reason for the present results. Another obvious difference to the study by Qian et al. (2012) is the reduced number of T2 lags in the present study. However, we have shown before that reducing T2 lags might slightly reduce the AB deficit but does not affect the overall pattern of performance (Kranczioch et al., 2003). In line with this, qualitatively, T2 performance at lags 2 and 7 in our study and the study by Qian et al. (2012) is very comparable. Moreover, for unmasked fearful and disgust faces preceding the RSVP stream, the emotion of the face has been reported to affect T2 performance in an AB paradigm with also only two T2 lags (Vermeulen et al., 2009). Though it cannot be ruled out at this stage, it is difficult to conceive that reducing T2 lags would specifically abolish the awareness-modulation of the emotional manipulation. Also different from the present study, Qian et al. (2012) used Japanese and Caucasian faces of unreported proportion for supposedly Chinese participants, while our stimulus set consisted of Caucasian faces from a Dutch database and our participants were German. An in-group advantage exists for the recognition of emotional faces from the same social group (Elfenbein and Ambady, 2002), suggesting that an effect of emotion should have, if anything, been stronger in the present study, where participants and face stimuli were Caucasian. On the other hand, research suggests that faces from negatively valanced social groups attract more attention when people search for negative emotion such as fear (Otten, 2016). It is impossible to tell whether this effect is of any relevance for the results of the study of Qian et al. (2012). If it was it could be expected to affect the effect of the emotional faces. Testing this possibility is however beyond the scope of the present study. Thus, no unequivocal reason for not finding a modulation of temporal attention by aware or unaware fearful faces in the present experiments can be identified. Future research should therefore focus on pinning down the pre-requisites of an awareness-dependent modulation of temporal attention by emotional faces, for instance in a setup varying social group of the participants and social group and valence of the social group of the face stimuli.

### Slight Impairment in Temporal Attention for Fearful Faces

Though we did not observe the expected awareness-dependent modulation of the effect of fearful faces on the AB, in one of the experiments fearful faces were associated with reduced T2 performance, irrespective of T2 lag and masking condition. This effect remained significant in the combined analysis of the two experiments, though numerically it was rather small (*M* = 2% in the combined analysis). The finding of a detrimental effect of fearful faces on the AB is in line with the ideas that negative emotional stimuli can restrict the attentional focus (Fenske and Eastwood, 2003; Dhinakaran et al., 2013) and that the AB is associated with a too narrow attentional focus (Olivers and Nieuwenhuis, 2006). Though numerically much smaller, it is also in line with Experiment 1 of Qian et al. (2012) and with the finding of Vermeulen et al. (2009) that RSVP-preceding unmasked fearful faces reduce T2 performance at lag 2 compared to disgust faces. The effect seems however specific to faces, as an AB study that instead of faces used positive, negative and neutral pictures of the International Affective Picture System (Lang et al., 2005) found that while positive pictures reduced the temporal attention deficit, negative pictures had no effect (Olivers and Nieuwenhuis, 2006). That in the present study the effect of emotion was independent of masking condition suggests that fearful faces elicit a similar attention modulation irrespective of the level of awareness.

### Practice Effects

An effect of time on task was evident for T2 performance. When data of both experiments were pooled for subsequent analysis, a modulation of the time on task effect by lag became apparent: the effect was larger for lag 2 than for lag 7, corresponding to a reduction of the AB. The literature is conflicting on whether the AB is reduced because of task practice. A number of studies found that for a moderate amount of practice of the AB task, no lag-specific practice effects occur in addition to the overall, lag-unspecific practice effect (Seiffert and Di Lollo, 1997; Choi et al., 2012; Nakatani et al., 2012; Kranczioch and Thorne, 2013), yet some evidence to the contrary exists as well (Taatgen et al., 2009). Studies specifically investigating whether the AB can be reduced or even abolished by practice indicate that strong learning effects can be observed, but only if the experimental setup fosters strong expectations regarding the temporal position of T2 during practice, achievable by fixing the position of T1 in the RSVP stream and at the same time having a single T2 lag in the AB period (Choi et al., 2012; Tang et al., 2013). Yet without these strong expectations and when taking care of ceiling effects, in spite of some reduction in AB size with training, the AB appears to be a robust phenomenon (Enns et al., 2017). Our data indicate that some lag-specific practice effect can also occur in setups with only moderate restrictions on T2 lag and T1 position, a setup typical for studies that include neurophysiological measures such as the electroencephalogram (Kessler et al., 2005; Janson et al., 2014; Kranczioch and Thorne, 2015; Śmigasiewicz et al., 2015) or the BOLD response of fMRI imaging (Kranczioch et al., 2007; De Martino et al., 2009; Hein et al., 2009; Lim et al., 2009). However, in the present study, the lag-specificity of the practice effect became only significant when, by pooling the data of two experiments, sample size, and statistical power increased. Hence, for a moderate amount of practice the learning effect seems small. Nevertheless, its presence should be taken into consideration when designing studies with restriction on T1 and in particular T2 positions.

## Conclusion

In conclusion, in two experiments the present study failed to conceptually replicate the previously reported awareness-dependent modulation of the AB by fearful faces (Qian et al., 2012). Results however indicate that irrespective of the awareness level, fearful faces can slightly but significantly reduce temporal attention performance compared to neutral faces. Thus, before studying the neural correlates of an awareness-dependent emotional modulation of the AB, research will have to focus on pinning down the pre-requisites of observing such modulation, including the role of the social group when using faces as stimulus material.

## Data Availability Statement

The datasets generated for this study are available on request to the corresponding author.

## Ethics Statement

The studies involving human participants were reviewed and approved by the Kommission für Forschungsfolgenabschätzung und Ethik (EK). The patients/participants provided their written informed consent to participate in this study.

## Author Contributions

JG and CK designed the study, analyzed the data, and wrote the manuscript. JG collected the data.

## Conflict of Interest

The authors declare that the research was conducted in the absence of any commercial or financial relationships that could be construed as a potential conflict of interest.

## References

[B1] Choi H. Chang L. H. Shibata K. Sasaki Y. Watanabe T. (2012). Resetting capacity limitations revealed by long-lasting elimination of attentional blink through training. *Proc. Natl. Acad. Sci. U.S.A.* 109 12242–12247. 10.1073/pnas.1203972109 22778408 PMC3409736

[B2] Chun M. M. Potter M. C. (1995). A two-stage model for multiple target detection in rapid serial visual presentation. *J. Exp. Psychol. Hum. Percept. Perform.* 21 109–127. 10.1037/0096-1523.21.1.109 7707027

[B3] De Martino B. Kalisch R. Rees G. Dolan R. J. (2009). Enhanced processing of threat stimuli under limited attentional resources. *Cereb. Cortex* 19 127–133. 10.1093/cercor/bhn062 18448453 PMC2638742

[B4] Dhinakaran J. De Vos M. Thorne J. D. Braun N. Janson J. Kranczioch C. (2013). Tough doughnuts: affect and the modulation of attention. *Front. Hum. Neurosci.* 7:876. 10.3389/fnhum.2013.00876 24391570 PMC3866654

[B5] Elfenbein H. A. Ambady N. (2002). On the universality and cultural specificity of emotion recognition: a meta-analysis. *Psychol. Bull.* 128 203–235. 10.1037/0033-2909.128.2.203 11931516

[B6] Enns J. T. Kealong P. Tichon J. G. Visser T. A. W. (2017). Training and the attentional blink: raising the ceiling does not remove the limits. *Atten. Percept. Psychophys.* 79 2257–2274. 10.3758/s13414-017-13919 28741100

[B7] Faul F. Erdfelder E. Lang A. G. Buchner A. (2007). G^∗^Power 3: a flexible statistical power analysis program for the social, behavioral, and biomedical sciences. *Behav. Res. Methods* 39 175–191. 10.3758/BF03193146 17695343

[B8] Fenske M. J. Eastwood J. D. (2003). Modulation of Focused attention by faces expressing emotion: evidence from flanker tasks. *Emotion* 3 327–334. 10.1037/1528-3542.3.4.327 14674827

[B9] Hein G. Alink A. Kleinschmidt A. Müller N. G. (2009). The attentional blink modulates activity in the early visual cortex. *J. Cogn. Neurosci.* 21 197–206. 10.1162/jocn.2008.21026 18510438

[B10] Janson J. De Vos M. Thorne J. D. Kranczioch C. (2014). Endogenous and rapid serial visual presentation-induced alpha band oscillations in the attentional blink. *J. Cogn. Neurosci.* 26 1454–1468. 10.1162/jocn_a_00551 24392903

[B11] Jasp Team (2019). JASP (Version 0.11.0)[Computer software]. 10.1162/jocn_a_00551 24392903

[B12] Kessler K. Schmitz F. Gross J. Hommel B. Shapiro K. Schnitzler A. (2005). Cortical mechanisms of attention in time: neural correlates of the Lag-1-sparing phenomenon. *Eur. J. Neurosci.* 21 2563–2574. 10.1111/j.1460-9568.2005.04063.x 15932614

[B13] Kranczioch C. Debener S. Engel A. K. (2003). Event-related potential correlates of the attentional blink phenomenon. *Cogn. Brain Res.* 17 177–187. 10.1016/s0926-6410(03)00092-2 12763203

[B14] Kranczioch C. Debener S. Schwarzbach J. Goebel R. Engel A. K. (2005). Neural correlates of conscious perception in the attentional blink. *Neuroimage* 24 704–714. 10.1016/j.neuroimage.2004.09.024 15652305

[B15] Kranczioch C. Debener S. Schwarzbach J. Goebel R. Engel A. K. (2007). Neural correlates of conscious perception in the attentional blink. *Neuroimage* 24 704–714. 10.1016/j.neuroimage.2004.09.024 15652305

[B16] Kranczioch C. Thorne J. D. (2013). Simultaneous and preceding sounds enhance rapid visual targets: evidence from the attentional blink. *Adv. Cogn. Psychol.* 9 130–142. 10.2478/v10053-008-0139-4 24155861 PMC3783938

[B17] Kranczioch C. Thorne J. D. (2015). The beneficial effects of sounds on attentional blink performance: an ERP study. *NeuroImage* 117 429–438. 10.1016/j.neuroimage.2015.05.055 26021217

[B18] Lang P. J. Bradley M. M. Cuthbert B. N. (2005). *International Affective Picture System (IAPS): Digitized Photographs, Instruction Manual, and Affective Ratings (Technical Report A-6).* Gainesville, FL: University of Florida.

[B19] Langner O. Dotsch R. Bijlstra G. Wigboldus D. H. J. Hawk S. T. Knippenberg A. F. M. (2010). Presentation and validation of the radboud faces database. *J. Cogn. Emot.* 24 1377–1388. 10.1080/02699930903485076

[B20] Lim L. Padmala S. Pessoa L. (2009). Segregating the significant from the mundane on a moment-to-moment basis via direct and indirect amygdala contributions. *PNAS* 106 16841–16846. 10.1073/pnas.0904551106 19805383 PMC2757860

[B21] Macmillan N. (1986). The psychophysics of subliminal perception. *Behav. Brain Sci.* 9 38–39. 10.1017/S0140525X00021427

[B22] Marois R. Yi D. J. Chun M. M. (2004). The neural fate of consciously perceived and missed events in the attentional blink. *Neuron* 41 465–472. 10.1016/s0896-6273(04)00012-1 14766184

[B23] Matsumoto D. Ekman P. (1988). *Japanese and Caucasian Facial Expressions of Emotion (JACFEE) and Neutral Faces (JACNeuF).* San Francisco, CA: San Francisco State University.

[B24] Meng M. Potter M. C. (2011). An attentional blink for nontargets? *Atten. Percept. Psychophys.* 73 440–446. 10.3758/s13414-010-0052-z 21264713

[B25] Nakatani C. Baijal S. Leeeuwen C. (2012). Curbing the attentional blink: practice keeps the mind’s eye open. *Neurocomputing* 84 13–22. 10.1016/j.neucom.2011.12.022

[B26] Oliver L. Dotsch R. Bijlstra G. Wigboldus D. H. J. Hawk S. T. van Knippenberg A. (2010). Presentation and validation of the radboud faces database. *Cogn. Emot.* 8 1377–1388. 10.1080/02699930903485076

[B27] Olivers C. N. Nieuwenhuis S. (2006). The beneficial effects of additional task load, positive affect, and instruction on the attentional blink. *J. Expo. Psychol. Hum. Percept. Perform.* 32 364–379. 10.1037/0096-1523.32.2.364 16634676

[B28] Otten M. (2016). Race guides attention in visual search. *PLoS One* 11:e0149158. 10.1371/journal.pone.0149158 26900957 PMC4763053

[B29] Pessoa L. Adolphs R. (2010). Emotion processing and the amygdala: from a ‘low road’ to ‘many roads’ of evaluating biological significance. *Nat. Rev. Neurosci.* 11 773–783. 10.1038/nrn2920 20959860 PMC3025529

[B30] Qian W. Meng Q. Chen L. Zhou K. (2012). Emotional modulation of the attentional blink is awareness- dependent. *PLoS One* 7:e43694. 10.1371/journal.pone.0043694 23029507 PMC3459896

[B31] Raymond J. E. Shapiro K. L. Arnell K. M. (1992). Temporary suppression of visual processing in an RSVP task: an attentional blink? *J. Expo. Psychol. Hum. Percept. Perform.* 18 849–860. 10.1037/0096-1523.18.3.849 1500880

[B32] Rouder J. N. Morey R. D. Speckman P. L. Province J. M. (2012). Default Bayes factors for ANOVA designs. *J. Math. Psychol.* 56 356–374. 10.1016/j.jmp.2012.08.001

[B33] Seiffert A. E. Di Lollo V. (1997). Low-level masking in the attentional blink. *J. Exp. Psychol. Hum. Percept. Perform.* 23 1061–1073. 10.1037/0096-1523.23.4.1061

[B34] Shapiro K. L. Raymond J. E. Arnell K. M. (1994). Attention to visual pattern information produces the attentional blink in rapid serial visual presentation. *J. Exp. Psychol. Hum. Percept. Perform.* 20 357–371. 10.1037/0096-1523.20.2.357 8189198

[B35] Śmigasiewicz K. Asanowicz D. Westphal N. Verleger R. (2015). Bias for the left visual field in rapid serial visual presentation: effects of additional salient cues suggest a critical role of attention. *J. Cogn. Neurosci.* 27 266–279. 10.1162/jocn_a_0071 25203275

[B36] Taatgen N. A. Juvina I. Schipper M. Borst J. P. Martens S. (2009). Too much control can hurt: a threaded cognition model of the attentional blink. *Cogn. Psychol.* 59 1–29. 10.1016/j.cogpsych.2008.12.002 19217086

[B37] Tang M. F. Badcock D. R. Visser T. A. (2013). Training and the attentional blink: limits overcome, or expectations raised? *Psychol. Bull. Rev.* 21 406–411. 10.3758/s13423-013-0491-3 23884691

[B38] Vermeulen N. Godefroid J. Mermillod M. (2009). Emotional modulation of attention: fear increases but disgust reduces the attentional blink. *PLoS One* 4:e7924. 10.1371/journal.pone.0007924 19936235 PMC2775630

[B39] Vogel E. K. Luck S. J. Shapiro K. L. (1998). Electrophysiological evidence for a postperceptual locus of suppression during the attentional blink. *J. Exp. Psychol. Hum. Percept. Perform.* 24 1656–1674. 10.1037//0096-1523.24.6.1656 9861716

