# Supplementary material for: No Evidence for an Awareness-Dependent Emotional Modulation of the Attentional Blink
Source: Front Psychol. 2019 Oct 25;10:2422. doi: 10.3389/fpsyg.2019.02422 (PMC6842977; doi:10.3389/fpsyg.2019.02422)
Supplement: Supplementary file 1 [file Table_1.pdf]

Results Experiment 1 - Supplementary Table S1

Bayesian Repeated Measures ANOVA

Model Comparison

Models	P(M)	P(M|data)	BF_M	BF_{10}	error %
T2 Lag + Gruppe + T2 Lag ∗ Gruppe	0.006	0.264	59.566	1.000	
T2 Lag + emotion + Gruppe + T2 Lag ∗ Gruppe	0.006	0.148	28.786	0.560	2.730
T2 Lag + masking + Gruppe + T2 Lag ∗ Gruppe + masking ∗ Gruppe	0.006	0.096	17.689	0.365	11.671
T2 Lag + masking + Gruppe + T2 Lag ∗ Gruppe	0.006	0.064	11.442	0.244	9.480
T2 Lag + masking + emotion + Gruppe + T2 Lag ∗ Gruppe + masking ∗ Gruppe	0.006	0.044	7.666	0.167	2.966
T2 Lag + emotion + Gruppe + T2 Lag ∗ Gruppe + emotion ∗ Gruppe	0.006	0.037	6.443	0.141	4.050
T2 Lag + emotion + Gruppe + T2 Lag ∗ emotion + T2 Lag ∗ Gruppe	0.006	0.037	6.376	0.140	3.337
T2 Lag + masking + Gruppe + T2 Lag ∗ masking + T2 Lag ∗ Gruppe + masking ∗ Gruppe	0.006	0.035	5.963	0.131	5.026
T2 Lag + masking + emotion + Gruppe + T2 Lag ∗ Gruppe	0.006	0.034	5.845	0.129	4.183
T2 Lag + masking + Gruppe + T2 Lag ∗ masking + T2 Lag ∗ Gruppe	0.006	0.025	4.322	0.096	7.862
T2 Lag + masking + Gruppe + T2 Lag ∗ masking + T2 Lag ∗ Gruppe + masking ∗ Gruppe + T2 Lag ∗ masking ∗ Gruppe	0.006	0.020	3.446	0.077	4.525
T2 Lag + masking + emotion + Gruppe + T2 Lag ∗ masking + T2 Lag ∗ Gruppe + masking ∗ Gruppe	0.006	0.019	3.151	0.071	3.622
T2 Lag + masking + emotion + Gruppe + T2 Lag ∗ masking + T2 Lag ∗ Gruppe	0.006	0.015	2.570	0.058	13.127
T2 Lag + masking + emotion + Gruppe + T2 Lag ∗ masking + T2 Lag ∗ Gruppe + masking ∗ Gruppe + T2 Lag ∗ masking ∗ Gruppe	0.006	0.013	2.197	0.049	10.049
T2 Lag + masking + emotion + Gruppe + masking ∗ emotion + T2 Lag ∗ Gruppe + masking ∗ Gruppe	0.006	0.013	2.135	0.048	3.818
T2 Lag + masking + emotion + Gruppe + T2 Lag ∗ Gruppe + masking ∗ Gruppe + emotion ∗ Gruppe	0.006	0.013	2.132	0.048	13.494
T2 Lag + masking + emotion + Gruppe + T2 Lag ∗ emotion + T2 Lag ∗ Gruppe + masking ∗ Gruppe	0.006	0.013	2.119	0.048	4.701
T2 Lag + emotion + Gruppe + T2 Lag ∗ emotion + T2 Lag ∗ Gruppe + emotion ∗ Gruppe	0.006	0.009	1.494	0.034	6.956
T2 Lag + masking + emotion + Gruppe + masking ∗ emotion + T2 Lag ∗ Gruppe	0.006	0.009	1.466	0.033	3.394
T2 Lag + masking + emotion + Gruppe + T2 Lag ∗ emotion + T2 Lag ∗ Gruppe	0.006	0.008	1.408	0.032	6.406
T2 Lag + masking + emotion + Gruppe + T2 Lag ∗ Gruppe + emotion ∗ Gruppe	0.006	0.008	1.389	0.031	6.177
T2 Lag + masking + emotion + Gruppe + T2 Lag ∗ masking + masking ∗ emotion + T2 Lag ∗ Gruppe + masking ∗ Gruppe	0.006	0.007	1.118	0.025	22.536
T2 Lag + masking + emotion + Gruppe + T2 Lag ∗ masking + T2 Lag ∗ Gruppe + masking ∗ Gruppe + emotion ∗ Gruppe	0.006	0.005	0.824	0.019	9.072
T2 Lag + masking + emotion + Gruppe + T2 Lag ∗ masking + T2 Lag ∗ emotion + T2 Lag ∗ Gruppe + masking ∗ Gruppe	0.006	0.005	0.790	0.018	4.261
T2 Lag + masking + emotion + Gruppe + T2 Lag ∗ masking + masking ∗ emotion + T2 Lag ∗ Gruppe + masking ∗ Gruppe + T2 Lag ∗ masking ∗ Gruppe	0.006	0.004	0.665	0.015	21.329
T2 Lag + masking + emotion + Gruppe + masking ∗ emotion + T2 Lag ∗ Gruppe + masking ∗ Gruppe + emotion ∗ Gruppe	0.006	0.004	0.639	0.015	7.272
T2 Lag + masking + emotion + Gruppe + T2 Lag ∗ masking + T2 Lag ∗ emotion + T2 Lag ∗ Gruppe	0.006	0.004	0.595	0.014	13.278
T2 Lag + masking + emotion + Gruppe + T2 Lag ∗ masking + masking ∗ emotion + T2 Lag ∗ Gruppe	0.006	0.004	0.591	0.013	5.274
T2 Lag + masking + emotion + Gruppe + T2 Lag ∗ masking + T2 Lag ∗ Gruppe + emotion ∗ Gruppe	0.006	0.004	0.586	0.013	9.570
T2 Lag + masking + emotion + Gruppe + T2 Lag ∗ emotion + T2 Lag ∗ Gruppe + masking ∗ Gruppe + emotion ∗ Gruppe	0.006	0.003	0.572	0.013	13.858
T2 Lag + masking + emotion + Gruppe + T2 Lag ∗ emotion + masking ∗ emotion + T2 Lag ∗ Gruppe + masking ∗ Gruppe	0.006	0.003	0.512	0.012	3.878
T2 Lag + masking + emotion + Gruppe + T2 Lag ∗ masking + T2 Lag ∗ Gruppe + masking ∗ Gruppe + emotion ∗ Gruppe + T2 Lag ∗ masking ∗ Gruppe	0.006	0.003	0.511	0.012	6.642
T2 Lag + masking + emotion + Gruppe + T2 Lag ∗ masking + T2 Lag ∗ emotion + T2 Lag ∗ Gruppe + masking ∗ Gruppe + T2 Lag ∗ masking ∗ Gruppe	0.006	0.003	0.494	0.011	5.689
T2 Lag + emotion + Gruppe + T2 Lag ∗ emotion + T2 Lag ∗ Gruppe + emotion ∗ Gruppe + T2 Lag ∗ emotion ∗ Gruppe	0.006	0.003	0.445	0.010	3.908
T2 Lag + masking + emotion + Gruppe + masking ∗ emotion + T2 Lag ∗ Gruppe + emotion ∗ Gruppe	0.006	0.002	0.382	0.009	9.129
T2 Lag + masking + emotion + Gruppe + T2 Lag ∗ emotion + T2 Lag ∗ Gruppe + emotion ∗ Gruppe	0.006	0.002	0.370	0.008	6.883
T2 Lag + masking + emotion + Gruppe + T2 Lag ∗ emotion + masking ∗ emotion + T2 Lag ∗ Gruppe	0.006	0.002	0.365	0.008	6.044
T2 Lag + masking + emotion + Gruppe + T2 Lag ∗ masking + T2 Lag ∗ emotion + masking ∗ emotion + T2 Lag ∗ Gruppe + masking ∗ Gruppe	0.006	0.001	0.224	0.005	4.687
T2 Lag + masking + emotion + Gruppe + T2 Lag ∗ masking + T2 Lag ∗ emotion + T2 Lag ∗ Gruppe + masking ∗ Gruppe + emotion ∗ Gruppe	0.006	0.001	0.208	0.005	7.279
T2 Lag + masking + emotion + Gruppe + T2 Lag ∗ masking + masking ∗ emotion + T2 Lag ∗ Gruppe + masking ∗ Gruppe + emotion ∗ Gruppe	0.006	0.001	0.205	0.005	4.653
T2 Lag + masking + emotion + Gruppe + T2 Lag ∗ masking + T2 Lag ∗ emotion + T2 Lag ∗ Gruppe + emotion ∗ Gruppe	0.006	9.967e -4	0.166	0.004	7.915
T2 Lag + masking + emotion + Gruppe + T2 Lag ∗ emotion + T2 Lag ∗ Gruppe + masking ∗ Gruppe + emotion ∗ Gruppe + T2 Lag ∗ emotion ∗ Gruppe	0.006	9.480e -4	0.158	0.004	6.419
T2 Lag + masking + emotion + Gruppe + masking ∗ emotion + T2 Lag ∗ Gruppe + masking ∗ Gruppe + emotion ∗ Gruppe + masking ∗ emotion ∗ Gruppe	0.006	9.335e -4	0.155	0.004	4.111
T2 Lag + masking + emotion + Gruppe + T2 Lag ∗ masking + T2 Lag ∗ emotion + masking ∗ emotion + T2 Lag ∗ Gruppe	0.006	9.311e -4	0.155	0.004	11.138
T2 Lag + masking + emotion + Gruppe + T2 Lag ∗ masking + masking ∗ emotion + T2 Lag ∗ Gruppe + emotion ∗ Gruppe	0.006	9.230e -4	0.153	0.003	6.678
T2 Lag + masking + emotion + Gruppe + T2 Lag ∗ masking + T2 Lag ∗ emotion + masking ∗ emotion + T2 Lag ∗ Gruppe + masking ∗ Gruppe + T2 Lag ∗ masking ∗ Gruppe	0.006	8.527e -4	0.142	0.003	9.871
T2 Lag + masking + emotion + Gruppe + T2 Lag ∗ masking + T2 Lag ∗ emotion + T2 Lag ∗ Gruppe + masking ∗ Gruppe + emotion ∗ Gruppe + T2 Lag ∗ masking ∗ Gruppe	0.006	8.251e -4	0.137	0.003	9.048
T2 Lag + masking + emotion + Gruppe + T2 Lag ∗ masking + masking ∗ emotion + T2 Lag ∗ Gruppe + masking ∗ Gruppe + emotion ∗ Gruppe + T2 Lag ∗ masking ∗ Gruppe	0.006	8.234e -4	0.137	0.003	12.279
T2 Lag + masking + emotion + Gruppe + T2 Lag ∗ emotion + masking ∗ emotion + T2 Lag ∗ Gruppe + masking ∗ Gruppe + emotion ∗ Gruppe	0.006	7.891e -4	0.131	0.003	11.204
T2 Lag + masking + emotion + Gruppe + T2 Lag ∗ emotion + T2 Lag ∗ Gruppe + emotion ∗ Gruppe + T2 Lag ∗ emotion ∗ Gruppe	0.006	5.868e -4	0.097	0.002	5.930
T2 Lag + masking + emotion + Gruppe + T2 Lag ∗ emotion + masking ∗ emotion + T2 Lag ∗ Gruppe + emotion ∗ Gruppe	0.006	5.650e -4	0.094	0.002	8.084
T2 Lag + masking + emotion + Gruppe + T2 Lag ∗ masking + T2 Lag ∗ emotion + masking ∗ emotion + T2 Lag ∗ Gruppe + masking ∗ Gruppe + T2 Lag ∗ masking ∗ emotion	0.006	5.416e -4	0.090	0.002	12.775
T2 Lag + masking + emotion + Gruppe + T2 Lag ∗ masking + T2 Lag ∗ emotion + T2 Lag ∗ Gruppe + masking ∗ Gruppe + emotion ∗ Gruppe + T2 Lag ∗ emotion ∗ Gruppe	0.006	5.074e -4	0.084	0.002	12.879
T2 Lag + masking + emotion + Gruppe + T2 Lag ∗ masking + masking ∗ emotion + T2 Lag ∗ Gruppe + masking ∗ Gruppe + emotion ∗ Gruppe + masking ∗ emotion ∗ Gruppe	0.006	4.558e -4	0.076	0.002	8.462
T2 Lag + masking + emotion + Gruppe + T2 Lag ∗ masking + T2 Lag ∗ emotion + masking ∗ emotion + T2 Lag ∗ Gruppe + masking ∗ Gruppe + emotion ∗ Gruppe	0.006	3.454e -4	0.057	0.001	9.048
T2 Lag + masking + emotion + Gruppe + T2 Lag ∗ masking + T2 Lag ∗ emotion + masking ∗ emotion + T2 Lag ∗ Gruppe + T2 Lag ∗ masking ∗ emotion	0.006	2.900e -4	0.048	0.001	4.134
T2 Lag + masking + emotion + Gruppe + T2 Lag ∗ masking + T2 Lag ∗ emotion + masking ∗ emotion + T2 Lag ∗ Gruppe + emotion ∗ Gruppe	0.006	2.883e -4	0.048	0.001	13.906
T2 Lag + masking + emotion + Gruppe + T2 Lag ∗ masking + T2 Lag ∗ emotion + masking ∗ emotion + T2 Lag ∗ Gruppe + masking ∗ Gruppe + T2 Lag ∗ masking ∗ emotion + T2 Lag ∗ masking ∗ Gruppe	0.006	2.714e -4	0.045	0.001	6.040
T2 Lag + masking + emotion + Gruppe + T2 Lag ∗ masking + T2 Lag ∗ emotion + T2 Lag ∗ Gruppe + emotion ∗ Gruppe + T2 Lag ∗ emotion ∗ Gruppe	0.006	2.551e -4	0.042	9.661e -4	5.057
T2 Lag + masking + emotion + Gruppe + T2 Lag ∗ emotion + masking ∗ emotion + T2 Lag ∗ Gruppe + masking ∗ Gruppe + emotion ∗ Gruppe + masking ∗ emotion ∗ Gruppe	0.006	2.524e -4	0.042	9.557e -4	9.206
T2 Lag + masking + emotion + Gruppe + T2 Lag ∗ emotion + masking ∗ emotion + T2 Lag ∗ Gruppe + masking ∗ Gruppe + emotion ∗ Gruppe + T2 Lag ∗ emotion ∗ Gruppe	0.006	2.315e -4	0.038	8.766e -4	4.883
T2 Lag + masking + emotion + Gruppe + T2 Lag ∗ masking + masking ∗ emotion + T2 Lag ∗ Gruppe + masking ∗ Gruppe + emotion ∗ Gruppe + T2 Lag ∗ masking ∗ Gruppe + masking ∗ emotion ∗ Gruppe	0.006	2.290e -4	0.038	8.672e -4	7.149
T2 Lag + masking + emotion + Gruppe + T2 Lag ∗ masking + T2 Lag ∗ emotion + T2 Lag ∗ Gruppe + masking ∗ Gruppe + emotion ∗ Gruppe + T2 Lag ∗ masking ∗ Gruppe + T2 Lag ∗ emotion ∗ Gruppe	0.006	2.239e -4	0.037	8.478e -4	9.028
T2 Lag + masking + emotion + Gruppe + T2 Lag ∗ masking + T2 Lag ∗ emotion + masking ∗ emotion + T2 Lag ∗ Gruppe + masking ∗ Gruppe + emotion ∗ Gruppe + T2 Lag ∗ masking ∗ Gruppe	0.006	2.132e -4	0.035	8.073e -4	6.686
T2 Lag + masking + emotion + Gruppe + T2 Lag ∗ emotion + masking ∗ emotion + T2 Lag ∗ Gruppe + emotion ∗ Gruppe + T2 Lag ∗ emotion ∗ Gruppe	0.006	1.958e -4	0.033	7.413e -4	15.713
T2 Lag + masking + emotion + Gruppe + T2 Lag ∗ masking + T2 Lag ∗ emotion + masking ∗ emotion + T2 Lag ∗ Gruppe + masking ∗ Gruppe + emotion ∗ Gruppe + T2 Lag ∗ masking ∗ emotion	0.006	1.326e -4	0.022	5.020e -4	17.567
T2 Lag + masking + emotion + Gruppe + T2 Lag ∗ masking + T2 Lag ∗ emotion + masking ∗ emotion + T2 Lag ∗ Gruppe + masking ∗ Gruppe + emotion ∗ Gruppe + T2 Lag ∗ emotion ∗ Gruppe	0.006	1.097e -4	0.018	4.155e -4	8.137
T2 Lag + masking + emotion + Gruppe + T2 Lag ∗ masking + T2 Lag ∗ emotion + masking ∗ emotion + T2 Lag ∗ Gruppe + masking ∗ Gruppe + emotion ∗ Gruppe + masking ∗ emotion ∗ Gruppe	0.006	1.063e -4	0.018	4.025e -4	6.270
T2 Lag + masking + emotion + Gruppe + T2 Lag ∗ masking + T2 Lag ∗ emotion + masking ∗ emotion + T2 Lag ∗ Gruppe + emotion ∗ Gruppe + T2 Lag ∗ masking ∗ emotion	0.006	8.332e -5	0.014	3.155e -4	9.957
T2 Lag + masking + emotion + Gruppe + T2 Lag ∗ masking + T2 Lag ∗ emotion + masking ∗ emotion + T2 Lag ∗ Gruppe + masking ∗ Gruppe + emotion ∗ Gruppe + T2 Lag ∗ masking ∗ Gruppe + T2 Lag ∗ emotion ∗ Gruppe	0.006	7.586e -5	0.013	2.873e -4	17.871
T2 Lag + masking + emotion + Gruppe + T2 Lag ∗ masking + T2 Lag ∗ emotion + masking ∗ emotion + T2 Lag ∗ Gruppe + masking ∗ Gruppe + emotion ∗ Gruppe + T2 Lag ∗ masking ∗ emotion + T2 Lag ∗ masking ∗ Gruppe	0.006	7.395e -5	0.012	2.800e -4	9.794
T2 Lag + masking + emotion + Gruppe + T2 Lag ∗ emotion + masking ∗ emotion + T2 Lag ∗ Gruppe + masking ∗ Gruppe + emotion ∗ Gruppe + T2 Lag ∗ emotion ∗ Gruppe + masking ∗ emotion ∗ Gruppe	0.006	7.388e -5	0.012	2.798e -4	6.479
T2 Lag + masking + emotion + Gruppe + T2 Lag ∗ masking + T2 Lag ∗ emotion + masking ∗ emotion + T2 Lag ∗ Gruppe + masking ∗ Gruppe + emotion ∗ Gruppe + T2 Lag ∗ masking ∗ Gruppe + masking ∗ emotion ∗ Gruppe	0.006	6.825e -5	0.011	2.584e -4	17.026
T2 Lag + masking + emotion + Gruppe + T2 Lag ∗ masking + T2 Lag ∗ emotion + masking ∗ emotion + T2 Lag ∗ Gruppe + emotion ∗ Gruppe + T2 Lag ∗ emotion ∗ Gruppe	0.006	6.301e -5	0.010	2.386e -4	4.687
T2 Lag + masking + emotion + Gruppe + T2 Lag ∗ masking + T2 Lag ∗ emotion + masking ∗ emotion + T2 Lag ∗ Gruppe + masking ∗ Gruppe + emotion ∗ Gruppe + T2 Lag ∗ masking ∗ emotion + T2 Lag ∗ emotion ∗ Gruppe	0.006	3.780e -5	0.006	1.431e -4	13.176
T2 Lag + masking + emotion + Gruppe + T2 Lag ∗ masking + T2 Lag ∗ emotion + masking ∗ emotion + T2 Lag ∗ Gruppe + masking ∗ Gruppe + emotion ∗ Gruppe + T2 Lag ∗ masking ∗ emotion + masking ∗ emotion ∗ Gruppe	0.006	3.420e -5	0.006	1.295e -4	9.184
T2 Lag + masking + emotion + Gruppe + T2 Lag ∗ masking + T2 Lag ∗ emotion + masking ∗ emotion + T2 Lag ∗ Gruppe + masking ∗ Gruppe + emotion ∗ Gruppe + T2 Lag ∗ emotion ∗ Gruppe + masking ∗ emotion ∗ Gruppe	0.006	3.296e -5	0.005	1.248e -4	6.567
T2 Lag + masking + emotion + Gruppe + T2 Lag ∗ masking + T2 Lag ∗ emotion + masking ∗ emotion + T2 Lag ∗ Gruppe + masking ∗ Gruppe + emotion ∗ Gruppe + T2 Lag ∗ masking ∗ emotion + T2 Lag ∗ masking ∗ Gruppe + masking ∗ emotion ∗ Gruppe	0.006	2.501e -5	0.004	9.472e -5	14.758
T2 Lag + masking + emotion + Gruppe + T2 Lag ∗ masking + T2 Lag ∗ emotion + masking ∗ emotion + T2 Lag ∗ Gruppe + emotion ∗ Gruppe + T2 Lag ∗ masking ∗ emotion + T2 Lag ∗ emotion ∗ Gruppe	0.006	2.406e -5	0.004	9.112e -5	6.894
T2 Lag	0.006	2.287e -5	0.004	8.659e -5	2.040
T2 Lag + masking + emotion + Gruppe + T2 Lag ∗ masking + T2 Lag ∗ emotion + masking ∗ emotion + T2 Lag ∗ Gruppe + masking ∗ Gruppe + emotion ∗ Gruppe + T2 Lag ∗ masking ∗ emotion + T2 Lag ∗ masking ∗ Gruppe + T2 Lag ∗ emotion ∗ Gruppe	0.006	1.971e -5	0.003	7.462e -5	7.686
T2 Lag + masking + emotion + Gruppe + T2 Lag ∗ masking + T2 Lag ∗ emotion + masking ∗ emotion + T2 Lag ∗ Gruppe + masking ∗ Gruppe + emotion ∗ Gruppe + T2 Lag ∗ masking ∗ Gruppe + T2 Lag ∗ emotion ∗ Gruppe + masking ∗ emotion ∗ Gruppe	0.006	1.770e -5	0.003	6.701e -5	7.182
T2 Lag + Gruppe	0.006	1.599e -5	0.003	6.055e -5	2.381

Model Comparison

Models	P(M)	P(M|data)	BF_M	BF_{10}	error %

Note. All models include subject

T2 Lag + emotion	0.006	1.112e-5	0.002	4.211e-5	3.134
T2 Lag + masking + emotion + Gruppe + T2 Lag ✻ masking + T2 Lag ✻ emotion + masking ✻ emotion + T2 Lag ✻ Gruppe + masking ✻ Gruppe + emotion ✻ Gruppe + T2 Lag ✻ masking ✻ emotion + T2 Lag ✻ emotion ✻ Gruppe + masking ✻ emotion ✻ Gruppe	0.006	1.044e-5	0.002	3.954e-5	6.610
T2 Lag + masking + emotion + Gruppe + T2 Lag ✻ masking + T2 Lag ✻ emotion + masking ✻ emotion + T2 Lag ✻ Gruppe + masking ✻ Gruppe + emotion ✻ Gruppe + T2 Lag ✻ masking ✻ emotion + T2 Lag ✻ masking ✻ Gruppe + T2 Lag ✻ emotion ✻ Gruppe + masking ✻ emotion ✻ Gruppe	0.006	1.010e-5	0.002	3.826e-5	20.152
T2 Lag + emotion + Gruppe	0.006	7.451e-6	0.001	2.822e-5	3.146
T2 Lag + masking	0.006	4.756e-6	7.895e-4	1.801e-5	2.293
T2 Lag + masking + Gruppe	0.006	3.917e-6	6.502e-4	1.483e-5	17.376
T2 Lag + masking + Gruppe + masking ✻ Gruppe	0.006	3.684e-6	6.116e-4	1.395e-5	4.295
T2 Lag + emotion + T2 Lag ✻ emotion	0.006	2.623e-6	4.354e-4	9.932e-6	4.133
T2 Lag + masking + emotion + Gruppe + T2 Lag ✻ masking + T2 Lag ✻ emotion + masking ✻ emotion + T2 Lag ✻ Gruppe + masking ✻ Gruppe + emotion ✻ Gruppe + T2 Lag ✻ masking ✻ emotion + T2 Lag ✻ masking ✻ Gruppe + T2 Lag ✻ emotion ✻ Gruppe + masking ✻ emotion ✻ Gruppe + T2 Lag ✻ masking ✻ emotion ✻ Gruppe	0.006	2.522e-6	4.187e-4	9.551e-6	11.077
T2 Lag + masking + emotion	0.006	2.171e-6	3.604e-4	8.222e-6	2.616
T2 Lag + masking + emotion + Gruppe	0.006	1.972e-6	3.274e-4	7.468e-6	16.205
T2 Lag + masking + T2 Lag ✻ masking	0.006	1.834e-6	3.045e-4	6.946e-6	3.248
T2 Lag + emotion + Gruppe + T2 Lag ✻ emotion	0.006	1.749e-6	2.903e-4	6.623e-6	2.686
T2 Lag + emotion + Gruppe + emotion ✻ Gruppe	0.006	1.731e-6	2.874e-4	6.556e-6	2.860
T2 Lag + masking + emotion + Gruppe + masking ✻ Gruppe	0.006	1.721e-6	2.856e-4	6.515e-6	3.481
T2 Lag + masking + Gruppe + T2 Lag ✻ masking + masking ✻ Gruppe	0.006	1.370e-6	2.274e-4	5.188e-6	6.601
T2 Lag + masking + Gruppe + T2 Lag ✻ masking	0.006	1.347e-6	2.235e-4	5.100e-6	5.829
T2 Lag + masking + emotion + T2 Lag ✻ masking	0.006	8.794e-7	1.460e-4	3.330e-6	6.170
T2 Lag + masking + emotion + Gruppe + T2 Lag ✻ masking + masking ✻ Gruppe	0.006	6.362e-7	1.056e-4	2.409e-6	3.677
T2 Lag + masking + emotion + masking ✻ emotion	0.006	5.806e-7	9.638e-5	2.199e-6	2.851
T2 Lag + masking + emotion + Gruppe + T2 Lag ✻ masking	0.006	5.687e-7	9.440e-5	2.154e-6	3.539
T2 Lag + masking + emotion + T2 Lag ✻ emotion	0.006	5.261e-7	8.733e-5	1.992e-6	2.742
T2 Lag + emotion + Gruppe + T2 Lag ✻ emotion + emotion ✻ Gruppe	0.006	4.937e-7	8.195e-5	1.869e-6	8.134
T2 Lag + masking + emotion + Gruppe + masking ✻ emotion + masking ✻ Gruppe	0.006	4.477e-7	7.432e-5	1.695e-6	4.885
T2 Lag + masking + emotion + Gruppe + T2 Lag ✻ emotion + masking ✻ Gruppe	0.006	4.377e-7	7.265e-5	1.657e-6	5.597
T2 Lag + masking + emotion + Gruppe + T2 Lag ✻ emotion	0.006	4.124e-7	6.847e-5	1.562e-6	7.222
T2 Lag + masking + emotion + Gruppe + masking ✻ Gruppe + emotion ✻ Gruppe	0.006	4.028e-7	6.686e-5	1.525e-6	6.432
T2 Lag + masking + emotion + Gruppe + emotion ✻ Gruppe	0.006	3.958e-7	6.570e-5	1.499e-6	5.821
T2 Lag + masking + emotion + Gruppe + masking ✻ emotion	0.006	3.949e-7	6.555e-5	1.495e-6	3.177
T2 Lag + masking + emotion + Gruppe + T2 Lag ✻ masking + masking ✻ Gruppe + emotion ✻ Gruppe	0.006	3.661e-7	6.077e-5	1.386e-6	60.959
T2 Lag + masking + emotion + T2 Lag ✻ masking + masking ✻ emotion	0.006	2.366e-7	3.928e-5	8.961e-7	5.398
T2 Lag + masking + emotion + T2 Lag ✻ masking + T2 Lag ✻ emotion	0.006	2.058e-7	3.415e-5	7.791e-7	3.986
T2 Lag + masking + emotion + Gruppe + T2 Lag ✻ masking + masking ✻ emotion + masking ✻ Gruppe	0.006	1.985e-7	3.295e-5	7.517e-7	19.325
T2 Lag + masking + emotion + Gruppe + T2 Lag ✻ masking + emotion ✻ Gruppe	0.006	1.848e-7	3.068e-5	6.999e-7	17.655
T2 Lag + masking + emotion + Gruppe + T2 Lag ✻ masking + T2 Lag ✻ emotion + masking ✻ Gruppe	0.006	1.607e-7	2.668e-5	6.087e-7	5.282
T2 Lag + masking + emotion + Gruppe + T2 Lag ✻ masking + masking ✻ emotion	0.006	1.476e-7	2.451e-5	5.591e-7	3.685
T2 Lag + masking + emotion + Gruppe + T2 Lag ✻ emotion + masking ✻ emotion	0.006	1.400e-7	2.324e-5	5.301e-7	28.756
T2 Lag + masking + emotion + T2 Lag ✻ emotion + masking ✻ emotion	0.006	1.383e-7	2.296e-5	5.237e-7	3.213
T2 Lag + masking + emotion + Gruppe + T2 Lag ✻ masking + T2 Lag ✻ emotion	0.006	1.338e-7	2.221e-5	5.067e-7	4.214
T2 Lag + masking + emotion + Gruppe + T2 Lag ✻ emotion + masking ✻ emotion + masking ✻ Gruppe	0.006	1.079e-7	1.790e-5	4.085e-7	3.976
T2 Lag + masking + emotion + Gruppe + masking ✻ emotion + masking ✻ Gruppe + emotion ✻ Gruppe	0.006	1.045e-7	1.735e-5	3.957e-7	3.690
T2 Lag + masking + emotion + Gruppe + T2 Lag ✻ emotion + masking ✻ Gruppe + emotion ✻ Gruppe	0.006	9.840e-8	1.633e-5	3.726e-7	4.870
T2 Lag + masking + emotion + Gruppe + T2 Lag ✻ emotion + emotion ✻ Gruppe	0.006	9.549e-8	1.585e-5	3.616e-7	6.000
T2 Lag + masking + emotion + Gruppe + masking ✻ emotion + emotion ✻ Gruppe	0.006	9.164e-8	1.521e-5	3.470e-7	3.751
T2 Lag + masking + emotion + T2 Lag ✻ masking + T2 Lag ✻ emotion + masking ✻ emotion	0.006	5.340e-8	8.865e-6	2.022e-7	3.422
T2 Lag + masking + emotion + Gruppe + T2 Lag ✻ masking + T2 Lag ✻ emotion + masking ✻ emotion + masking ✻ Gruppe	0.006	4.445e-8	7.379e-6	1.683e-7	6.873
T2 Lag + masking + emotion + Gruppe + T2 Lag ✻ masking + masking ✻ emotion + masking ✻ Gruppe + emotion ✻ Gruppe	0.006	4.376e-8	7.265e-6	1.657e-7	7.445
T2 Lag + masking + emotion + Gruppe + T2 Lag ✻ masking + T2 Lag ✻ emotion + emotion ✻ Gruppe	0.006	3.853e-8	6.397e-6	1.459e-7	11.839
T2 Lag + masking + emotion + Gruppe + T2 Lag ✻ masking + masking ✻ emotion + emotion ✻ Gruppe	0.006	3.754e-8	6.232e-6	1.422e-7	7.696
T2 Lag + masking + emotion + Gruppe + T2 Lag ✻ masking + T2 Lag ✻ emotion + masking ✻ Gruppe + emotion ✻ Gruppe	0.006	3.616e-8	6.003e-6	1.369e-7	4.524
T2 Lag + masking + emotion + Gruppe + T2 Lag ✻ masking + T2 Lag ✻ emotion + masking ✻ emotion	0.006	3.581e-8	5.945e-6	1.356e-7	4.089
T2 Lag + masking + emotion + Gruppe + masking ✻ emotion + masking ✻ Gruppe + emotion ✻ Gruppe + masking ✻ emotion ✻ Gruppe	0.006	3.370e-8	5.595e-6	1.276e-7	4.979
T2 Lag + masking + emotion + Gruppe + T2 Lag ✻ emotion + masking ✻ emotion + masking ✻ Gruppe + emotion ✻ Gruppe	0.006	2.589e-8	4.297e-6	9.803e-8	5.060
T2 Lag + masking + emotion + Gruppe + T2 Lag ✻ emotion + masking ✻ emotion + emotion ✻ Gruppe	0.006	2.480e-8	4.116e-6	9.390e-8	4.715
T2 Lag + masking + emotion + T2 Lag ✻ masking + T2 Lag ✻ emotion + masking ✻ emotion + T2 Lag ✻ masking ✻ emotion	0.006	1.765e-8	2.930e-6	6.683e-8	4.048
T2 Lag + masking + emotion + Gruppe + T2 Lag ✻ masking + masking ✻ emotion + masking ✻ Gruppe + emotion ✻ Gruppe + masking ✻ emotion ✻ Gruppe	0.006	1.761e-8	2.924e-6	6.669e-8	10.842
T2 Lag + masking + emotion + Gruppe + T2 Lag ✻ masking + T2 Lag ✻ emotion + masking ✻ emotion + masking ✻ Gruppe + T2 Lag ✻ masking ✻ emotion	0.006	1.414e-8	2.347e-6	5.353e-8	6.501
T2 Lag + masking + emotion + Gruppe + T2 Lag ✻ masking + T2 Lag ✻ emotion + masking ✻ emotion + T2 Lag ✻ masking ✻ emotion	0.006	1.334e-8	2.214e-6	5.052e-8	7.916
T2 Lag + masking + emotion + Gruppe + T2 Lag ✻ masking + T2 Lag ✻ emotion + masking ✻ emotion + emotion ✻ Gruppe	0.006	9.755e-9	1.619e-6	3.694e-8	6.797
T2 Lag + masking + emotion + Gruppe + T2 Lag ✻ masking + T2 Lag ✻ emotion + masking ✻ emotion + masking ✻ Gruppe + emotion ✻ Gruppe	0.006	9.561e-9	1.587e-6	3.621e-8	5.393
T2 Lag + masking + emotion + Gruppe + T2 Lag ✻ emotion + masking ✻ emotion + masking ✻ Gruppe + emotion ✻ Gruppe + masking ✻ emotion ✻ Gruppe	0.006	8.133e-9	1.350e-6	3.080e-8	8.354
T2 Lag + masking + emotion + Gruppe + T2 Lag ✻ masking + T2 Lag ✻ emotion + masking ✻ emotion + emotion ✻ Gruppe + T2 Lag ✻ masking ✻ emotion	0.006	3.341e-9	5.545e-7	1.265e-8	8.941
T2 Lag + masking + emotion + Gruppe + T2 Lag ✻ masking + T2 Lag ✻ emotion + masking ✻ emotion + masking ✻ Gruppe + emotion ✻ Gruppe + T2 Lag ✻ masking ✻ emotion	0.006	3.251e-9	5.397e-7	1.231e-8	5.431
T2 Lag + masking + emotion + Gruppe + T2 Lag ✻ masking + T2 Lag ✻ emotion + masking ✻ emotion + masking ✻ Gruppe + emotion ✻ Gruppe + masking ✻ emotion ✻ Gruppe	0.006	2.866e-9	4.757e-7	1.085e-8	4.910
T2 Lag + masking + emotion + Gruppe + T2 Lag ✻ masking + T2 Lag ✻ emotion + masking ✻ emotion + masking ✻ Gruppe + emotion ✻ Gruppe + T2 Lag ✻ masking ✻ emotion + masking ✻ emotion ✻ Gruppe	0.006	1.026e-9	1.703e-7	3.886e-9	6.416
Null model (incl. subject)	0.006	1.518e-29	2.520e-27	5.748e-29	1.767
Gruppe	0.006	7.861e-30	1.305e-27	2.977e-29	1.953
emotion	0.006	4.149e-30	6.887e-28	1.571e-29	2.992
masking	0.006	2.787e-30	4.627e-28	1.055e-29	1.978
emotion + Gruppe	0.006	2.281e-30	3.787e-28	8.639e-30	5.902
masking + Gruppe	0.006	1.491e-30	2.474e-28	5.645e-30	3.348
masking + emotion	0.006	7.434e-31	1.234e-28	2.815e-30	2.680
masking + Gruppe + masking ✻ Gruppe	0.006	6.485e-31	1.076e-28	2.456e-30	4.179
emotion + Gruppe + emotion ✻ Gruppe	0.006	5.855e-31	9.719e-29	2.217e-30	13.568
masking + emotion + Gruppe	0.006	3.940e-31	6.541e-29	1.492e-30	3.786
masking + emotion + masking ✻ emotion	0.006	1.875e-31	3.113e-29	7.101e-31	4.384
masking + emotion + Gruppe + masking ✻ Gruppe	0.006	1.792e-31	2.974e-29	6.784e-31	5.694
masking + emotion + Gruppe + masking ✻ emotion	0.006	9.503e-32	1.578e-29	3.599e-31	3.684
masking + emotion + Gruppe + emotion ✻ Gruppe	0.006	9.113e-32	1.513e-29	3.451e-31	3.247
masking + emotion + Gruppe + masking ✻ emotion + masking ✻ Gruppe	0.006	4.258e-32	7.069e-30	1.613e-31	4.931
masking + emotion + Gruppe + masking ✻ Gruppe + emotion ✻ Gruppe	0.006	4.058e-32	6.736e-30	1.537e-31	4.535
masking + emotion + Gruppe + masking ✻ emotion + emotion ✻ Gruppe	0.006	2.395e-32	3.976e-30	9.071e-32	7.256
masking + emotion + Gruppe + masking ✻ emotion + masking ✻ Gruppe + emotion ✻ Gruppe	0.006	1.153e-32	1.914e-30	4.365e-32	18.122
masking + emotion + Gruppe + masking ✻ emotion + masking ✻ Gruppe + emotion ✻ Gruppe + masking ✻ emotion ✻ Gruppe	0.006	2.870e-33	4.764e-31	1.087e-32	3.784

Note. All models include subject

Analysis of Effects

Effects	P(incl)	P(incl|data)	BF_{incl}
T2 Lag	0.114	7.983e-5	2.170e+24
masking	0.114	0.118	0.237
emotion	0.114	0.273	0.540
Gruppe	0.114	3.427e-5	0.714
T2 Lag ✻ masking	0.299	0.130	0.401
T2 Lag ✻ emotion	0.299	0.098	0.252
masking ✻ emotion	0.299	0.056	0.279
Gruppe ✻ T2 Lag	0.299	0.947	19998.619
Gruppe ✻ masking	0.299	0.266	1.421
Gruppe ✻ emotion	0.299	0.099	0.256
T2 Lag ✻ masking ✻ emotion	0.114	0.002	0.349
Gruppe ✻ T2 Lag ✻ masking	0.114	0.047	0.622
Gruppe ✻ T2 Lag ✻ emotion	0.114	0.006	0.296
Gruppe ✻ masking ✻ emotion	0.114	0.002	0.284
Gruppe ✻ T2 Lag ✻ masking ✻ emotion	0.006	2.522e-6	0.250

Note. Compares models that contain the effect to equivalent models stripped of the effect. Higher-order interactions are excluded. Analysis suggested by Sebastiaan Mathôt.
