# Supplementary material for: No Evidence for an Awareness-Dependent Emotional Modulation of the Attentional Blink
Source: Front Psychol. 2019 Oct 25;10:2422. doi: 10.3389/fpsyg.2019.02422 (PMC6842977; doi:10.3389/fpsyg.2019.02422)
Supplement: Supplementary file 2 [file Table_2.pdf]

Results Experiment 2 - Supplementary Table S2

Bayesian Repeated Measures ANOVA

Model Comparison

Models	P(M)	P(M|data)	BF M	BF 10	error %
T2 Lag + masking + Gruppe + masking * Gruppe	0.006	0.319	77.650	1.000	
T2 Lag	0.006	0.082	14.766	0.256	2.712
T2 Lag + masking + Gruppe + T2 Lag * masking + masking * Gruppe	0.006	0.078	14.039	0.245	6.001
T2 Lag + masking + Gruppe + T2 Lag * Gruppe + masking * Gruppe	0.006	0.076	13.700	0.239	4.551
T2 Lag + masking + emotion + Gruppe + masking * Gruppe	0.006	0.068	12.019	0.212	4.897
T2 Lag + masking + Gruppe + T2 Lag * masking + T2 Lag * Gruppe + masking * Gruppe + T2 Lag * masking * Gruppe	0.006	0.044	7.576	0.137	8.846
T2 Lag + Gruppe	0.006	0.043	7.466	0.135	2.833
T2 Lag + masking	0.006	0.023	3.993	0.074	3.000
T2 Lag + masking + emotion + Gruppe + T2 Lag * emotion + masking * Gruppe	0.006	0.020	3.419	0.063	3.985
T2 Lag + masking + Gruppe + T2 Lag * masking + T2 Lag * Gruppe + masking * Gruppe	0.006	0.018	2.962	0.055	6.745
T2 Lag + emotion	0.006	0.017	2.859	0.053	3.655
T2 Lag + masking + emotion + Gruppe + T2 Lag * masking + masking * Gruppe	0.006	0.016	2.636	0.049	6.872
T2 Lag + masking + emotion + Gruppe + masking * emotion + masking * Gruppe	0.006	0.015	2.593	0.048	4.384
T2 Lag + masking + emotion + Gruppe + T2 Lag * Gruppe + masking * Gruppe	0.006	0.015	2.470	0.046	3.487
T2 Lag + masking + emotion + Gruppe + masking * Gruppe + emotion * Gruppe	0.006	0.015	2.445	0.046	3.799
T2 Lag + masking + Gruppe	0.006	0.012	2.057	0.038	3.195
T2 Lag + Gruppe + T2 Lag * Gruppe	0.006	0.010	1.684	0.032	3.156
T2 Lag + emotion + Gruppe	0.006	0.009	1.558	0.029	4.629
T2 Lag + masking + emotion + Gruppe + T2 Lag * masking + T2 Lag * Gruppe + masking * Gruppe + T2 Lag * masking * Gruppe	0.006	0.009	1.439	0.027	6.178
T2 Lag + masking + T2 Lag * masking	0.006	0.006	0.961	0.018	3.875
T2 Lag + masking + emotion + Gruppe + T2 Lag * masking + T2 Lag * emotion + masking * Gruppe	0.006	0.005	0.905	0.017	11.486
T2 Lag + emotion + T2 Lag * emotion	0.006	0.005	0.848	0.016	3.683
T2 Lag + masking + emotion	0.006	0.005	0.830	0.016	4.767
T2 Lag + masking + emotion + Gruppe + T2 Lag * emotion + masking * Gruppe + emotion * Gruppe	0.006	0.005	0.826	0.016	6.857
T2 Lag + masking + emotion + Gruppe + T2 Lag * emotion + T2 Lag * Gruppe + masking * Gruppe	0.006	0.005	0.806	0.015	4.161
T2 Lag + masking + emotion + Gruppe + T2 Lag * emotion + masking * emotion + masking * Gruppe	0.006	0.005	0.795	0.015	4.570
T2 Lag + masking + emotion + Gruppe + T2 Lag * masking + T2 Lag * Gruppe + masking * Gruppe	0.006	0.005	0.778	0.015	13.832
T2 Lag + masking + emotion + Gruppe + masking * emotion + T2 Lag * Gruppe + masking * Gruppe	0.006	0.004	0.706	0.013	12.129
T2 Lag + masking + emotion + Gruppe + T2 Lag * masking + masking * emotion + masking * Gruppe	0.006	0.004	0.632	0.012	10.818
T2 Lag + masking + emotion + Gruppe + masking * emotion + masking * Gruppe + emotion * Gruppe	0.006	0.003	0.565	0.011	7.178
T2 Lag + masking + emotion + Gruppe + T2 Lag * masking + masking * Gruppe + emotion * Gruppe	0.006	0.003	0.549	0.010	4.253
T2 Lag + masking + emotion + Gruppe + T2 Lag * Gruppe + masking * Gruppe + emotion * Gruppe	0.006	0.003	0.543	0.010	4.343
T2 Lag + masking + Gruppe + T2 Lag * Gruppe	0.006	0.003	0.535	0.010	5.711
T2 Lag + masking + Gruppe + T2 Lag * masking	0.006	0.003	0.488	0.009	4.734
T2 Lag + emotion + Gruppe + T2 Lag * emotion	0.006	0.003	0.450	0.008	3.606
T2 Lag + masking + emotion + Gruppe	0.006	0.003	0.430	0.008	3.616
T2 Lag + masking + emotion + Gruppe + T2 Lag * masking + T2 Lag * emotion + T2 Lag * Gruppe + masking * Gruppe + T2 Lag * masking * Gruppe	0.006	0.003	0.419	0.008	5.938
T2 Lag + emotion + Gruppe + emotion * Gruppe	0.006	0.002	0.353	0.007	5.306
T2 Lag + emotion + Gruppe + T2 Lag * Gruppe	0.006	0.002	0.347	0.007	3.746
T2 Lag + masking + emotion + Gruppe + T2 Lag * masking + masking * emotion + T2 Lag * Gruppe + masking * Gruppe + T2 Lag * masking * Gruppe	0.006	0.002	0.320	0.006	6.423
T2 Lag + masking + emotion + Gruppe + T2 Lag * masking + T2 Lag * Gruppe + masking * Gruppe + emotion * Gruppe + T2 Lag * masking * Gruppe	0.006	0.002	0.283	0.005	5.093
T2 Lag + masking + emotion + Gruppe + masking * emotion + masking * Gruppe + emotion * Gruppe + masking * emotion * Gruppe	0.006	0.002	0.280	0.005	6.376
T2 Lag + masking + emotion + T2 Lag * emotion	0.006	0.002	0.252	0.005	4.340
T2 Lag + masking + emotion + Gruppe + T2 Lag * masking + T2 Lag * emotion + masking * emotion + T2 Lag * Gruppe + masking * Gruppe + T2 Lag * masking * Gruppe	0.006	0.002	0.250	0.005	52.251
T2 Lag + masking + emotion + Gruppe + T2 Lag * masking + T2 Lag * emotion + masking * Gruppe + emotion * Gruppe	0.006	0.001	0.199	0.004	8.557
T2 Lag + masking + emotion + T2 Lag * masking	0.006	0.001	0.193	0.004	5.954
T2 Lag + masking + emotion + Gruppe + T2 Lag * masking + T2 Lag * emotion + masking * emotion + masking * Gruppe	0.006	0.001	0.190	0.004	6.329
T2 Lag + masking + emotion + masking * emotion	0.006	0.001	0.188	0.004	4.380
T2 Lag + masking + emotion + Gruppe + T2 Lag * emotion + masking * emotion + T2 Lag * Gruppe + masking * Gruppe	0.006	0.001	0.187	0.004	4.921
T2 Lag + masking + emotion + Gruppe + T2 Lag * emotion + masking * emotion + masking * Gruppe + emotion * Gruppe	0.006	0.001	0.184	0.003	6.144
T2 Lag + masking + emotion + Gruppe + T2 Lag * emotion + T2 Lag * Gruppe + masking * Gruppe + emotion * Gruppe	0.006	0.001	0.181	0.003	6.165
T2 Lag + masking + emotion + Gruppe + T2 Lag * masking + T2 Lag * emotion + T2 Lag * Gruppe + masking * Gruppe	0.006	0.001	0.171	0.003	4.202
T2 Lag + masking + emotion + Gruppe + masking * emotion + T2 Lag * Gruppe + masking * Gruppe + emotion * Gruppe	0.006	8.377e -4	0.139	0.003	9.116
T2 Lag + masking + emotion + Gruppe + T2 Lag * masking + masking * emotion + T2 Lag * Gruppe + masking * Gruppe	0.006	8.256e -4	0.137	0.003	5.114
T2 Lag + masking + emotion + Gruppe + T2 Lag * Gruppe	0.006	8.033e -4	0.133	0.003	17.844
T2 Lag + masking + emotion + Gruppe + T2 Lag * masking + T2 Lag * Gruppe + masking * Gruppe + emotion * Gruppe	0.006	7.903e -4	0.131	0.002	4.612
T2 Lag + masking + emotion + Gruppe + T2 Lag * emotion	0.006	7.728e -4	0.128	0.002	3.666
T2 Lag + masking + emotion + Gruppe + T2 Lag * masking + masking * emotion + masking * Gruppe + emotion * Gruppe	0.006	7.171e -4	0.119	0.002	4.676
T2 Lag + emotion + Gruppe + T2 Lag * emotion + T2 Lag * Gruppe	0.006	6.950e -4	0.115	0.002	7.190
T2 Lag + emotion + Gruppe + T2 Lag * emotion + emotion * Gruppe	0.006	6.765e -4	0.112	0.002	4.803
T2 Lag + masking + Gruppe + T2 Lag * masking + T2 Lag * Gruppe	0.006	6.359e -4	0.106	0.002	3.513
T2 Lag + masking + emotion + Gruppe + emotion * Gruppe	0.006	6.012e -4	0.100	0.002	4.467
T2 Lag + masking + emotion + Gruppe + masking * emotion	0.006	5.959e -4	0.099	0.002	7.312
T2 Lag + masking + emotion + Gruppe + T2 Lag * masking + T2 Lag * emotion + T2 Lag * Gruppe + masking * Gruppe + emotion * Gruppe + T2 Lag * masking * Gruppe	0.006	5.780e -4	0.096	0.002	4.940
T2 Lag + masking + emotion + Gruppe + T2 Lag * masking	0.006	5.598e -4	0.093	0.002	4.346
T2 Lag + emotion + Gruppe + T2 Lag * Gruppe + emotion * Gruppe	0.006	4.924e -4	0.082	0.002	4.500
T2 Lag + masking + emotion + Gruppe + T2 Lag * emotion + masking * emotion + masking * Gruppe + emotion * Gruppe + masking * emotion * Gruppe	0.006	4.912e -4	0.082	0.002	4.831
T2 Lag + masking + emotion + Gruppe + T2 Lag * emotion + T2 Lag * Gruppe + masking * Gruppe + emotion * Gruppe + T2 Lag * emotion * Gruppe	0.006	4.713e -4	0.078	0.001	16.268
T2 Lag + masking + emotion + Gruppe + T2 Lag * masking + masking * emotion + T2 Lag * Gruppe + masking * Gruppe + emotion * Gruppe + T2 Lag * masking * Gruppe	0.006	4.329e -4	0.072	0.001	8.511
T2 Lag + masking + emotion + Gruppe + T2 Lag * masking + T2 Lag * emotion + masking * emotion + masking * Gruppe + T2 Lag * masking * emotion	0.006	3.947e -4	0.066	0.001	5.687
T2 Lag + masking + emotion + T2 Lag * masking + T2 Lag * emotion	0.006	3.772e -4	0.063	0.001	9.835
T2 Lag + masking + emotion + Gruppe + T2 Lag * masking + masking * emotion + masking * Gruppe + emotion * Gruppe + masking * emotion * Gruppe	0.006	3.749e -4	0.062	0.001	5.636
T2 Lag + masking + emotion + Gruppe + masking * emotion + T2 Lag * Gruppe + masking * Gruppe + emotion * Gruppe + masking * emotion * Gruppe	0.006	3.741e -4	0.062	0.001	6.603
T2 Lag + masking + emotion + T2 Lag * emotion + masking * emotion	0.006	3.555e -4	0.059	0.001	7.996
T2 Lag + masking + emotion + Gruppe + T2 Lag * masking + T2 Lag * emotion + masking * emotion + T2 Lag * Gruppe + masking * Gruppe	0.006	2.637e -4	0.044	8.275e -4	6.223
T2 Lag + masking + emotion + T2 Lag * masking + masking * emotion	0.006	2.593e -4	0.043	8.136e -4	6.009
T2 Lag + masking + emotion + Gruppe + T2 Lag * masking + masking * emotion + T2 Lag * Gruppe + masking * Gruppe + emotion * Gruppe + T2 Lag * masking * Gruppe + masking * emotion * Gruppe	0.006	2.556e -4	0.042	8.021e -4	11.395
T2 Lag + masking + emotion + Gruppe + T2 Lag * emotion + masking * emotion + T2 Lag * Gruppe + masking * Gruppe + emotion * Gruppe	0.006	2.503e -4	0.042	7.853e -4	5.573
T2 Lag + masking + emotion + Gruppe + T2 Lag * masking + T2 Lag * emotion + masking * emotion + masking * Gruppe + emotion * Gruppe	0.006	2.384e -4	0.040	7.480e -4	4.924
T2 Lag + masking + emotion + Gruppe + T2 Lag * masking + T2 Lag * emotion + T2 Lag * Gruppe + masking * Gruppe + emotion * Gruppe + T2 Lag * masking * Gruppe + T2 Lag * emotion * Gruppe	0.006	2.363e -4	0.039	7.415e -4	16.074
T2 Lag + masking + emotion + Gruppe + T2 Lag * masking + T2 Lag * emotion + T2 Lag * Gruppe + masking * Gruppe + emotion * Gruppe	0.006	2.359e -4	0.039	7.401e -4	4.403
T2 Lag + masking + emotion + Gruppe + T2 Lag * emotion + T2 Lag * Gruppe	0.006	2.007e -4	0.033	6.299e -4	7.040
T2 Lag + masking + emotion + Gruppe + T2 Lag * masking + T2 Lag * emotion	0.006	1.855e -4	0.031	5.820e -4	5.413
T2 Lag + masking + emotion + Gruppe + masking * emotion + T2 Lag * Gruppe	0.006	1.852e -4	0.031	5.812e -4	28.714
T2 Lag + masking + emotion + Gruppe + T2 Lag * masking + masking * emotion + T2 Lag * Gruppe + masking * Gruppe + emotion * Gruppe	0.006	1.787e -4	0.030	5.607e -4	5.611
T2 Lag + masking + emotion + Gruppe + T2 Lag * masking + T2 Lag * emotion + masking * emotion + T2 Lag * Gruppe + masking * Gruppe + T2 Lag * masking * emotion + T2 Lag * masking * Gruppe	0.006	1.756e -4	0.029	5.511e -4	5.273
T2 Lag + masking + emotion + Gruppe + T2 Lag * emotion + emotion * Gruppe	0.006	1.731e -4	0.029	5.432e -4	3.943
T2 Lag + masking + emotion + Gruppe + T2 Lag * emotion + masking * emotion	0.006	1.720e -4	0.029	5.397e -4	3.839
T2 Lag + emotion + Gruppe + T2 Lag * emotion + T2 Lag * Gruppe + emotion * Gruppe	0.006	1.470e -4	0.024	4.611e -4	4.275
T2 Lag + masking + emotion + Gruppe + T2 Lag * Gruppe + emotion * Gruppe	0.006	1.457e -4	0.024	4.572e -4	6.622
T2 Lag + masking + emotion + Gruppe + T2 Lag * masking + emotion * Gruppe	0.006	1.410e -4	0.023	4.425e -4	5.013
T2 Lag + masking + emotion + Gruppe + T2 Lag * masking + T2 Lag * emotion + masking * emotion + masking * Gruppe + emotion * Gruppe + masking * emotion * Gruppe	0.006	1.352e -4	0.022	4.242e -4	10.360
T2 Lag + masking + emotion + Gruppe + T2 Lag * masking + T2 Lag * emotion + masking * emotion + T2 Lag * Gruppe + masking * Gruppe + emotion * Gruppe + T2 Lag * masking * Gruppe + T2 Lag * emotion * Gruppe + masking * emotion * Gruppe	0.006	1.350e -4	0.022	4.235e -4	83.572
T2 Lag + masking + emotion + Gruppe + T2 Lag * masking + T2 Lag * emotion + masking * emotion + T2 Lag * Gruppe + masking * Gruppe + emotion * Gruppe + T2 Lag * masking * Gruppe	0.006	1.344e -4	0.022	4.219e -4	6.145
T2 Lag + masking + emotion + Gruppe + T2 Lag * masking + T2 Lag * Gruppe	0.006	1.326e -4	0.022	4.162e -4	4.806
T2 Lag + masking + emotion + Gruppe + masking * emotion + emotion * Gruppe	0.006	1.280e -4	0.021	4.015e -4	3.837
T2 Lag + masking + emotion + Gruppe + T2 Lag * masking + masking * emotion	0.006	1.275e -4	0.021	4.000e -4	4.166
T2 Lag + masking + emotion + Gruppe + T2 Lag * emotion + masking * emotion + T2 Lag * Gruppe + masking * Gruppe + emotion * Gruppe + masking * emotion * Gruppe	0.006	1.251e -4	0.021	3.925e -4	7.976
T2 Lag + masking + emotion + Gruppe + T2 Lag * masking + T2 Lag * emotion + masking * emotion + T2 Lag * Gruppe + masking * Gruppe + emotion * [illegible]	0.006	9.589e -5	0.016	3.009e -4	23.575

Model Comparison

Models P(M) P(M|data) BF M BF 10 error %

Gruppe + T2 Lag ∗ masking ∗ Gruppe + masking ∗ emotion ∗ Gruppe

Note. All models include subject

T2 Lag + masking + emotion + Gruppe + T2 Lag ∗ masking + T2 Lag ∗ emotion + masking ∗ emotion + masking ∗ Gruppe + emotion ∗ Gruppe + T2 Lag ∗ masking ∗ emotion	0.006	8.877e -5	0.015	2.785e -4	6.492
T2 Lag + masking + emotion + Gruppe + T2 Lag ∗ masking + T2 Lag ∗ emotion + masking ∗ emotion + T2 Lag ∗ Gruppe + masking ∗ Gruppe + T2 Lag ∗ masking ∗ emotion	0.006	8.859e -5	0.015	2.780e -4	7.658
T2 Lag + masking + emotion + Gruppe + T2 Lag ∗ masking + masking ∗ emotion + T2 Lag ∗ Gruppe + masking ∗ Gruppe + emotion ∗ Gruppe + masking ∗ emotion ∗ Gruppe	0.006	8.628e -5	0.014	2.707e -4	4.951
T2 Lag + masking + emotion + T2 Lag ∗ masking + T2 Lag ∗ emotion + masking ∗ emotion	0.006	8.204e -5	0.014	2.574e -4	6.947
T2 Lag + masking + emotion + Gruppe + T2 Lag ∗ emotion + masking ∗ emotion + T2 Lag ∗ Gruppe + masking ∗ Gruppe + emotion ∗ Gruppe + T2 Lag ∗ emotion ∗ Gruppe	0.006	8.029e -5	0.013	2.519e -4	6.998
T2 Lag + masking + emotion + Gruppe + T2 Lag ∗ masking + T2 Lag ∗ emotion + T2 Lag ∗ Gruppe + masking ∗ Gruppe + emotion ∗ Gruppe + T2 Lag ∗ emotion ∗ Gruppe	0.006	7.148e -5	0.012	2.243e -4	4.295
T2 Lag + masking + emotion + Gruppe + T2 Lag ∗ masking + T2 Lag ∗ emotion + masking ∗ emotion + T2 Lag ∗ Gruppe + masking ∗ Gruppe + emotion ∗ Gruppe + T2 Lag ∗ masking ∗ emotion + T2 Lag ∗ masking ∗ Gruppe	0.006	6.802e -5	0.011	2.134e -4	14.770
T2 Lag + masking + emotion + Gruppe + T2 Lag ∗ masking + T2 Lag ∗ emotion + masking ∗ emotion + T2 Lag ∗ Gruppe + masking ∗ Gruppe + emotion ∗ Gruppe	0.006	5.804e -5	0.010	1.821e -4	5.446
T2 Lag + emotion + Gruppe + T2 Lag ∗ emotion + T2 Lag ∗ Gruppe + emotion ∗ Gruppe + T2 Lag ∗ emotion ∗ Gruppe	0.006	5.173e -5	0.009	1.623e -4	7.166
T2 Lag + masking + emotion + Gruppe + T2 Lag ∗ masking + T2 Lag ∗ emotion + masking ∗ emotion	0.006	4.951e -5	0.008	1.554e -4	19.811
T2 Lag + masking + emotion + Gruppe + T2 Lag ∗ emotion + masking ∗ emotion + T2 Lag ∗ Gruppe	0.006	4.842e -5	0.008	1.519e -4	6.524
T2 Lag + masking + emotion + Gruppe + T2 Lag ∗ masking + T2 Lag ∗ emotion + emotion ∗ Gruppe	0.006	4.789e -5	0.008	1.503e -4	7.214
T2 Lag + masking + emotion + Gruppe + T2 Lag ∗ masking + T2 Lag ∗ emotion + masking ∗ emotion + masking ∗ Gruppe + emotion ∗ Gruppe + T2 Lag ∗ masking ∗ emotion + masking ∗ emotion ∗ Gruppe	0.006	4.483e -5	0.007	1.407e -4	8.408
T2 Lag + masking + emotion + Gruppe + T2 Lag ∗ masking + T2 Lag ∗ emotion + masking ∗ emotion + T2 Lag ∗ Gruppe + masking ∗ Gruppe + emotion ∗ Gruppe + T2 Lag ∗ masking ∗ Gruppe + T2 Lag ∗ emotion ∗ Gruppe	0.006	4.460e -5	0.007	1.399e -4	11.970
T2 Lag + masking + emotion + Gruppe + T2 Lag ∗ emotion + masking ∗ emotion + T2 Lag ∗ Gruppe + masking ∗ Gruppe + emotion ∗ Gruppe + T2 Lag ∗ emotion ∗ Gruppe + masking ∗ emotion ∗ Gruppe	0.006	4.310e -5	0.007	1.352e -4	12.105
T2 Lag + masking + emotion + Gruppe + T2 Lag ∗ emotion + T2 Lag ∗ Gruppe + emotion ∗ Gruppe	0.006	4.135e -5	0.007	1.297e -4	4.588
T2 Lag + masking + emotion + Gruppe + T2 Lag ∗ masking + T2 Lag ∗ emotion + T2 Lag ∗ Gruppe	0.006	3.967e -5	0.007	1.245e -4	4.090
T2 Lag + masking + emotion + Gruppe + T2 Lag ∗ emotion + masking ∗ emotion + emotion ∗ Gruppe	0.006	3.848e -5	0.006	1.207e -4	4.720
T2 Lag + masking + emotion + Gruppe + masking ∗ emotion + T2 Lag ∗ Gruppe + emotion ∗ Gruppe	0.006	3.643e -5	0.006	1.143e -4	6.666
T2 Lag + masking + emotion + Gruppe + T2 Lag ∗ masking + T2 Lag ∗ emotion + masking ∗ emotion + T2 Lag ∗ Gruppe + masking ∗ Gruppe + emotion ∗ Gruppe + masking ∗ emotion ∗ Gruppe	0.006	3.205e -5	0.005	1.006e -4	8.933
T2 Lag + masking + emotion + Gruppe + T2 Lag ∗ masking + masking ∗ emotion + emotion ∗ Gruppe	0.006	3.188e -5	0.005	1.000e -4	5.656
T2 Lag + masking + emotion + Gruppe + T2 Lag ∗ masking + masking ∗ emotion + T2 Lag ∗ Gruppe	0.006	3.078e -5	0.005	9.658e -5	4.944
T2 Lag + masking + emotion + Gruppe + T2 Lag ∗ masking + T2 Lag ∗ Gruppe + emotion ∗ Gruppe	0.006	2.872e -5	0.005	9.012e -5	3.826
T2 Lag + masking + emotion + T2 Lag ∗ masking + T2 Lag ∗ emotion + masking ∗ emotion + T2 Lag ∗ masking ∗ emotion	0.006	2.610e -5	0.004	8.190e -5	6.119
T2 Lag + masking + emotion + Gruppe + T2 Lag ∗ masking + T2 Lag ∗ emotion + masking ∗ emotion + T2 Lag ∗ Gruppe + masking ∗ Gruppe + emotion ∗ Gruppe + T2 Lag ∗ emotion ∗ Gruppe	0.006	2.185e -5	0.004	6.856e -5	10.880
T2 Lag + masking + emotion + Gruppe + T2 Lag ∗ masking + T2 Lag ∗ emotion + masking ∗ emotion + T2 Lag ∗ Gruppe + masking ∗ Gruppe + emotion ∗ Gruppe + T2 Lag ∗ masking ∗ emotion + T2 Lag ∗ masking ∗ Gruppe + masking ∗ emotion ∗ Gruppe	0.006	2.076e -5	0.003	6.515e -5	6.099
T2 Lag + masking + emotion + Gruppe + T2 Lag ∗ masking + T2 Lag ∗ emotion + masking ∗ emotion + T2 Lag ∗ Gruppe + masking ∗ Gruppe + emotion ∗ Gruppe + T2 Lag ∗ masking ∗ emotion	0.006	2.044e -5	0.003	6.415e -5	10.718
T2 Lag + masking + emotion + Gruppe + T2 Lag ∗ masking + T2 Lag ∗ emotion + masking ∗ emotion + T2 Lag ∗ Gruppe + masking ∗ Gruppe + emotion ∗ Gruppe + T2 Lag ∗ masking ∗ emotion + T2 Lag ∗ masking ∗ Gruppe + T2 Lag ∗ emotion ∗ Gruppe	0.006	1.838e -5	0.003	5.766e -5	14.799
T2 Lag + masking + emotion + Gruppe + T2 Lag ∗ masking + T2 Lag ∗ emotion + masking ∗ emotion + T2 Lag ∗ masking ∗ emotion	0.006	1.719e -5	0.003	5.393e -5	8.338
T2 Lag + masking + emotion + Gruppe + T2 Lag ∗ emotion + T2 Lag ∗ Gruppe + emotion ∗ Gruppe + T2 Lag ∗ emotion ∗ Gruppe	0.006	1.445e -5	0.002	4.533e -5	7.905
T2 Lag + masking + emotion + Gruppe + T2 Lag ∗ masking + T2 Lag ∗ emotion + masking ∗ emotion + emotion ∗ Gruppe	0.006	1.068e -5	0.002	3.351e -5	8.253
T2 Lag + masking + emotion + Gruppe + T2 Lag ∗ masking + T2 Lag ∗ emotion + masking ∗ emotion + T2 Lag ∗ Gruppe + masking ∗ Gruppe + emotion ∗ Gruppe + T2 Lag ∗ emotion ∗ Gruppe + masking ∗ emotion ∗ Gruppe	0.006	1.066e -5	0.002	3.344e -5	15.986
T2 Lag + masking + emotion + Gruppe + T2 Lag ∗ masking + T2 Lag ∗ emotion + T2 Lag ∗ Gruppe + emotion ∗ Gruppe	0.006	1.045e -5	0.002	3.280e -5	6.744
T2 Lag + masking + emotion + Gruppe + T2 Lag ∗ masking + T2 Lag ∗ emotion + masking ∗ emotion + T2 Lag ∗ Gruppe	0.006	1.034e -5	0.002	3.244e -5	10.936
T2 Lag + masking + emotion + Gruppe + T2 Lag ∗ masking + T2 Lag ∗ emotion + masking ∗ emotion + T2 Lag ∗ Gruppe + masking ∗ Gruppe + emotion ∗ Gruppe + T2 Lag ∗ masking ∗ emotion + masking ∗ emotion ∗ Gruppe	0.006	9.688e -6	0.002	3.040e -5	8.025
T2 Lag + masking + emotion + Gruppe + T2 Lag ∗ emotion + masking ∗ emotion + T2 Lag ∗ Gruppe + emotion ∗ Gruppe	0.006	9.686e -6	0.002	3.039e -5	7.016
T2 Lag + masking + emotion + Gruppe + T2 Lag ∗ masking + T2 Lag ∗ emotion + masking ∗ emotion + T2 Lag ∗ Gruppe + masking ∗ Gruppe + emotion ∗ Gruppe + T2 Lag ∗ masking ∗ emotion + T2 Lag ∗ masking ∗ Gruppe + T2 Lag ∗ emotion ∗ Gruppe + masking ∗ emotion ∗ Gruppe	0.006	7.924e -6	0.001	2.486e -5	8.249
T2 Lag + masking + emotion + Gruppe + T2 Lag ∗ masking + masking ∗ emotion + T2 Lag ∗ Gruppe + emotion ∗ Gruppe	0.006	7.912e -6	0.001	2.483e -5	7.162
T2 Lag + masking + emotion + Gruppe + T2 Lag ∗ masking + T2 Lag ∗ emotion + masking ∗ emotion + T2 Lag ∗ Gruppe + masking ∗ Gruppe + emotion ∗ Gruppe + T2 Lag ∗ masking ∗ emotion + T2 Lag ∗ emotion ∗ Gruppe	0.006	6.496e -6	0.001	2.038e -5	6.893
T2 Lag + masking + emotion + Gruppe + T2 Lag ∗ masking + T2 Lag ∗ emotion + masking ∗ emotion + T2 Lag ∗ Gruppe + masking ∗ Gruppe + emotion ∗ Gruppe + T2 Lag ∗ masking ∗ emotion + T2 Lag ∗ emotion ∗ Gruppe + masking ∗ emotion ∗ Gruppe	0.006	5.721e -6	9.498e -4	1.795e -5	34.618
T2 Lag + masking + emotion + Gruppe + T2 Lag ∗ masking + T2 Lag ∗ emotion + masking ∗ emotion + T2 Lag ∗ Gruppe + T2 Lag ∗ masking ∗ emotion	0.006	4.184e -6	6.945e -4	1.313e -5	8.999
T2 Lag + masking + emotion + Gruppe + T2 Lag ∗ masking + T2 Lag ∗ emotion + T2 Lag ∗ Gruppe + emotion ∗ Gruppe + T2 Lag ∗ emotion ∗ Gruppe	0.006	3.384e -6	5.617e -4	1.062e -5	8.792
T2 Lag + masking + emotion + Gruppe + T2 Lag ∗ masking + T2 Lag ∗ emotion + masking ∗ emotion + T2 Lag ∗ Gruppe + masking ∗ Gruppe + emotion ∗ Gruppe + T2 Lag ∗ masking ∗ emotion + T2 Lag ∗ masking ∗ Gruppe + T2 Lag ∗ emotion ∗ Gruppe + masking ∗ emotion ∗ Gruppe + T2 Lag ∗ masking ∗ emotion ∗ Gruppe	0.006	3.198e -6	5.309e -4	1.004e -5	15.588
T2 Lag + masking + emotion + Gruppe + T2 Lag ∗ masking + T2 Lag ∗ emotion + masking ∗ emotion + emotion ∗ Gruppe + T2 Lag ∗ masking ∗ emotion	0.006	3.148e -6	5.225e -4	9.877e -6	9.495
T2 Lag + masking + emotion + Gruppe + T2 Lag ∗ emotion + masking ∗ emotion + T2 Lag ∗ Gruppe + emotion ∗ Gruppe + T2 Lag ∗ emotion ∗ Gruppe	0.006	3.070e -6	5.097e -4	9.634e -6	5.395
T2 Lag + masking + emotion + Gruppe + T2 Lag ∗ masking + T2 Lag ∗ emotion + masking ∗ emotion + T2 Lag ∗ Gruppe + emotion ∗ Gruppe	0.006	2.664e -6	4.423e -4	8.360e -6	8.182
T2 Lag + masking + emotion + Gruppe + T2 Lag ∗ masking + T2 Lag ∗ emotion + masking ∗ emotion + T2 Lag ∗ Gruppe + emotion ∗ Gruppe + T2 Lag ∗ emotion ∗ Gruppe	0.006	7.718e -7	1.281e -4	2.422e -6	8.579
T2 Lag + masking + emotion + Gruppe + T2 Lag ∗ masking + T2 Lag ∗ emotion + masking ∗ emotion + T2 Lag ∗ Gruppe + emotion ∗ Gruppe + T2 Lag ∗ masking ∗ emotion	0.006	7.616e -7	1.264e -4	2.390e -6	6.113
T2 Lag + masking + emotion + Gruppe + T2 Lag ∗ masking + T2 Lag ∗ emotion + masking ∗ emotion + T2 Lag ∗ Gruppe + emotion ∗ Gruppe + T2 Lag ∗ masking ∗ emotion + T2 Lag ∗ emotion ∗ Gruppe	0.006	3.425e -7	5.686e -5	1.075e -6	22.645
Null model (incl. subject)	0.006	7.624e-15	1.266e-12	2.392e-14	2.554
Gruppe	0.006	3.560e-15	5.910e-13	1.117e-14	2.869
masking + Gruppe + masking ∗ Gruppe	0.006	3.424e-15	5.684e-13	1.074e-14	3.385
masking	0.006	1.736e-15	2.882e-13	5.448e-15	2.646
emotion	0.006	1.423e-15	2.362e-13	4.464e-15	2.691
masking + Gruppe	0.006	8.612e-16	1.430e-13	2.702e-15	5.006
emotion + Gruppe	0.006	6.799e-16	1.129e-13	2.133e-15	3.432
masking + emotion + Gruppe + masking ∗ Gruppe	0.006	6.392e-16	1.061e-13	2.006e-15	3.302
masking + emotion	0.006	3.324e-16	5.518e-14	1.043e-15	2.991
masking + emotion + Gruppe + masking ∗ Gruppe + emotion ∗ Gruppe	0.006	1.635e-16	2.714e-14	5.129e-16	4.501
masking + emotion + Gruppe	0.006	1.606e-16	2.666e-14	5.040e-16	3.791
masking + emotion + Gruppe + masking ∗ emotion + masking ∗ Gruppe	0.006	1.599e-16	2.654e-14	5.017e-16	4.764
emotion + Gruppe + emotion ∗ Gruppe	0.006	1.563e-16	2.595e-14	4.905e-16	5.037
masking + emotion + masking ∗ emotion	0.006	8.831e-17	1.466e-14	2.771e-16	10.659
masking + emotion + Gruppe + masking ∗ emotion	0.006	3.955e-17	6.565e-15	1.241e-16	8.258
masking + emotion + Gruppe + emotion ∗ Gruppe	0.006	3.490e-17	5.794e-15	1.095e-16	3.337
masking + emotion + Gruppe + masking ∗ emotion + masking ∗ Gruppe + emotion ∗ Gruppe	0.006	3.255e-17	5.403e-15	1.021e-16	3.849
masking + emotion + Gruppe + masking ∗ emotion + masking ∗ Gruppe + emotion ∗ Gruppe + masking ∗ emotion ∗ Gruppe	0.006	1.348e-17	2.237e-15	4.229e-17	4.981
masking + emotion + Gruppe + masking ∗ emotion + emotion ∗ Gruppe	0.006	8.079e-18	1.341e-15	2.535e-17	3.851

Note. All models include subject

Analysis of Effects

Effects	P(incl)	P(incl|data)	BF_{incl}
T2 Lag	0.114	0.620	2.933e+13
masking	0.114	0.051	0.290
emotion	0.114	0.150	0.209
Gruppe	0.114	0.075	0.527
T2 Lag ∗ masking	0.299	0.148	0.242
T2 Lag ∗ emotion	0.299	0.067	0.313
T2 Lag ∗ Gruppe	0.299	0.152	0.236
masking ∗ emotion	0.299	0.046	0.238
masking ∗ Gruppe	0.299	0.693	25.655
emotion ∗ Gruppe	0.299	0.044	0.218
T2 Lag ∗ masking ∗ emotion	0.114	0.001	0.252
T2 Lag ∗ masking ∗ Gruppe	0.114	0.062	2.395
T2 Lag ∗ emotion ∗ Gruppe	0.114	0.001	0.418
masking ∗ emotion ∗ Gruppe	0.114	0.004	0.511
T2 Lag ∗ masking ∗ emotion ∗ Gruppe	0.006	3.198e-6	0.404

Note. Compares models that contain the effect to equivalent models stripped of the effect. Higher-order interactions are excluded. Analysis suggested by Sebastiaan Mathôt.
