# Supplementary material for: No Evidence for an Awareness-Dependent Emotional Modulation of the Attentional Blink
Source: Front Psychol. 2019 Oct 25;10:2422. doi: 10.3389/fpsyg.2019.02422 (PMC6842977; doi:10.3389/fpsyg.2019.02422)
Supplement: Supplementary file 3 [file Table_3.pdf]

Results Experiments 1&2 joint analysis - Supplementary Table S3

Bayesian Repeated Measures ANOVA

Model Comparison

Models	P(M)	P(M|data)	BF_M	BF_{10}	error %
T2 Lag + masking + Gruppe + T2 Lag ∗ Gruppe + masking ∗ Gruppe	0.006	0.296	69.652	1.000	
T2 Lag + masking + Gruppe + T2 Lag ∗ masking + T2 Lag ∗ Gruppe + masking ∗ Gruppe + T2 Lag ∗ masking ∗ Gruppe	0.006	0.164	32.680	0.556	5.083
T2 Lag + masking + emotion + Gruppe + T2 Lag ∗ Gruppe + masking ∗ Gruppe	0.006	0.113	21.047	0.381	4.524
T2 Lag + masking + emotion + Gruppe + T2 Lag ∗ masking + T2 Lag ∗ Gruppe + masking ∗ Gruppe + T2 Lag ∗ masking ∗ Gruppe	0.006	0.074	13.227	0.250	9.114
T2 Lag + masking + Gruppe + T2 Lag ∗ masking + T2 Lag ∗ Gruppe + masking ∗ Gruppe	0.006	0.070	12.512	0.237	6.360
T2 Lag + Gruppe + T2 Lag ∗ Gruppe	0.006	0.043	7.448	0.145	3.365
T2 Lag + masking + emotion + Gruppe + T2 Lag ∗ emotion + T2 Lag ∗ Gruppe + masking ∗ Gruppe	0.006	0.026	4.480	0.089	4.916
T2 Lag + masking + emotion + Gruppe + T2 Lag ∗ masking + T2 Lag ∗ Gruppe + masking ∗ Gruppe	0.006	0.026	4.390	0.087	4.812
T2 Lag + masking + emotion + Gruppe + masking ∗ emotion + T2 Lag ∗ Gruppe + masking ∗ Gruppe	0.006	0.023	3.990	0.079	7.044
T2 Lag + masking + emotion + Gruppe + T2 Lag ∗ Gruppe + masking ∗ Gruppe + emotion ∗ Gruppe	0.006	0.019	3.202	0.064	5.104
T2 Lag + emotion + Gruppe + T2 Lag ∗ Gruppe	0.006	0.016	2.683	0.054	3.787
T2 Lag + masking + emotion + Gruppe + T2 Lag ∗ masking + T2 Lag ∗ emotion + T2 Lag ∗ Gruppe + masking ∗ Gruppe + T2 Lag ∗ masking ∗ Gruppe	0.006	0.014	2.385	0.048	5.988
T2 Lag + masking + emotion + Gruppe + T2 Lag ∗ masking + masking ∗ emotion + T2 Lag ∗ Gruppe + masking ∗ Gruppe + T2 Lag ∗ masking ∗ Gruppe	0.006	0.011	1.851	0.037	5.717
T2 Lag + masking + emotion + Gruppe + T2 Lag ∗ masking + T2 Lag ∗ Gruppe + masking ∗ Gruppe + emotion ∗ Gruppe + T2 Lag ∗ masking ∗ Gruppe	0.006	0.010	1.750	0.035	5.250
T2 Lag + masking + Gruppe + masking ∗ Gruppe	0.006	0.008	1.393	0.028	4.583
T2 Lag + masking + emotion + Gruppe + T2 Lag ∗ masking + T2 Lag ∗ emotion + T2 Lag ∗ Gruppe + masking ∗ Gruppe	0.006	0.006	0.945	0.019	5.023
T2 Lag + masking + Gruppe + T2 Lag ∗ Gruppe	0.006	0.005	0.893	0.018	3.529
T2 Lag + masking + emotion + Gruppe + T2 Lag ∗ masking + masking ∗ emotion + T2 Lag ∗ Gruppe + masking ∗ Gruppe	0.006	0.005	0.847	0.017	11.290
T2 Lag + masking + emotion + Gruppe + T2 Lag ∗ emotion + masking ∗ emotion + T2 Lag ∗ Gruppe + masking ∗ Gruppe	0.006	0.005	0.828	0.017	5.590
T2 Lag + masking + emotion + Gruppe + masking ∗ emotion + T2 Lag ∗ Gruppe + masking ∗ Gruppe + emotion ∗ Gruppe	0.006	0.005	0.765	0.016	11.429
T2 Lag + masking + emotion + Gruppe + T2 Lag ∗ emotion + T2 Lag ∗ Gruppe + masking ∗ Gruppe + emotion ∗ Gruppe	0.006	0.005	0.761	0.015	5.760
T2 Lag + masking + emotion + Gruppe + T2 Lag ∗ masking + T2 Lag ∗ Gruppe + masking ∗ Gruppe + emotion ∗ Gruppe	0.006	0.004	0.749	0.015	6.651
T2 Lag	0.006	0.004	0.745	0.015	3.060
T2 Lag + emotion + Gruppe + T2 Lag ∗ emotion + T2 Lag ∗ Gruppe	0.006	0.004	0.641	0.013	4.114
T2 Lag + masking + emotion + Gruppe + masking ∗ Gruppe	0.006	0.003	0.522	0.011	5.387
T2 Lag + masking + emotion + Gruppe + T2 Lag ∗ masking + T2 Lag ∗ emotion + T2 Lag ∗ Gruppe + masking ∗ Gruppe + emotion ∗ Gruppe + T2 Lag ∗ masking ∗ Gruppe	0.006	0.003	0.499	0.010	9.318
T2 Lag + emotion + Gruppe + T2 Lag ∗ Gruppe + emotion ∗ Gruppe	0.006	0.003	0.494	0.010	9.130
T2 Lag + masking + emotion + Gruppe + T2 Lag ∗ masking + T2 Lag ∗ emotion + masking ∗ emotion + T2 Lag ∗ Gruppe + masking ∗ Gruppe + T2 Lag ∗ masking ∗ Gruppe	0.006	0.003	0.446	0.009	6.094
T2 Lag + masking + emotion + Gruppe + T2 Lag ∗ Gruppe	0.006	0.002	0.337	0.007	4.724
T2 Lag + masking + emotion + Gruppe + T2 Lag ∗ masking + masking ∗ emotion + T2 Lag ∗ Gruppe + masking ∗ Gruppe + emotion ∗ Gruppe + T2 Lag ∗ masking ∗ Gruppe	0.006	0.002	0.337	0.007	8.678
T2 Lag + Gruppe	0.006	0.002	0.328	0.007	20.693
T2 Lag + masking + Gruppe + T2 Lag ∗ masking + masking ∗ Gruppe	0.006	0.002	0.287	0.006	4.486
T2 Lag + emotion	0.006	0.002	0.265	0.005	3.460
T2 Lag + masking + emotion + Gruppe + T2 Lag ∗ emotion + T2 Lag ∗ Gruppe + masking ∗ Gruppe + emotion ∗ Gruppe + T2 Lag ∗ emotion ∗ Gruppe	0.006	0.002	0.251	0.005	27.259
T2 Lag + masking + Gruppe + T2 Lag ∗ masking + T2 Lag ∗ Gruppe	0.006	0.001	0.233	0.005	10.539
T2 Lag + masking + emotion + Gruppe + T2 Lag ∗ masking + T2 Lag ∗ emotion + T2 Lag ∗ Gruppe + masking ∗ Gruppe + emotion ∗ Gruppe	0.006	0.001	0.211	0.004	14.932
T2 Lag + masking + emotion + Gruppe + T2 Lag ∗ masking + T2 Lag ∗ emotion + masking ∗ emotion + T2 Lag ∗ Gruppe + masking ∗ Gruppe	0.006	0.001	0.197	0.004	6.990
T2 Lag + masking + emotion + Gruppe + masking ∗ emotion + T2 Lag ∗ Gruppe + masking ∗ Gruppe + emotion ∗ Gruppe + masking ∗ emotion ∗ Gruppe	0.006	0.001	0.170	0.003	9.047
T2 Lag + masking + emotion + Gruppe + T2 Lag ∗ masking + masking ∗ Gruppe	0.006	0.001	0.166	0.003	23.450
T2 Lag + masking + emotion + Gruppe + T2 Lag ∗ emotion + masking ∗ emotion + T2 Lag ∗ Gruppe + masking ∗ Gruppe + emotion ∗ Gruppe	0.006	9.141e -4	0.152	0.003	5.798
T2 Lag + masking + emotion + Gruppe + T2 Lag ∗ masking + masking ∗ emotion + T2 Lag ∗ Gruppe + masking ∗ Gruppe + emotion ∗ Gruppe	0.006	8.238e -4	0.137	0.003	6.888
T2 Lag + masking + emotion + Gruppe + masking ∗ Gruppe + emotion ∗ Gruppe	0.006	7.322e -4	0.122	0.002	30.675
T2 Lag + masking + emotion + Gruppe + T2 Lag ∗ emotion + masking ∗ Gruppe	0.006	7.112e -4	0.118	0.002	5.106
T2 Lag + masking + emotion + Gruppe + masking ∗ emotion + masking ∗ Gruppe	0.006	6.918e -4	0.115	0.002	11.904
T2 Lag + emotion + Gruppe + T2 Lag ∗ emotion + T2 Lag ∗ Gruppe + emotion ∗ Gruppe	0.006	6.776e -4	0.113	0.002	5.761
T2 Lag + masking + emotion + Gruppe + T2 Lag ∗ masking + T2 Lag ∗ emotion + T2 Lag ∗ Gruppe + masking ∗ Gruppe + emotion ∗ Gruppe + T2 Lag ∗ masking ∗ Gruppe + T2 Lag ∗ emotion ∗ Gruppe	0.006	6.195e -4	0.103	0.002	11.653
T2 Lag + masking	0.006	5.918e -4	0.098	0.002	5.568
T2 Lag + masking + emotion + Gruppe + T2 Lag ∗ masking + T2 Lag ∗ emotion + masking ∗ emotion + T2 Lag ∗ Gruppe + masking ∗ Gruppe + T2 Lag ∗ masking ∗ emotion + T2 Lag ∗ masking ∗ Gruppe	0.006	5.598e -4	0.093	0.002	6.391
T2 Lag + masking + emotion + Gruppe + T2 Lag ∗ masking + masking ∗ emotion + T2 Lag ∗ Gruppe + masking ∗ Gruppe + emotion ∗ Gruppe + T2 Lag ∗ masking ∗ Gruppe + masking ∗ emotion ∗ Gruppe	0.006	5.207e -4	0.086	0.002	7.077
T2 Lag + emotion + Gruppe	0.006	5.123e -4	0.085	0.002	3.230
T2 Lag + masking + emotion + Gruppe + T2 Lag ∗ masking + T2 Lag ∗ Gruppe	0.006	4.793e -4	0.080	0.002	7.343
T2 Lag + masking + emotion + Gruppe + T2 Lag ∗ masking + T2 Lag ∗ emotion + masking ∗ emotion + T2 Lag ∗ Gruppe + masking ∗ Gruppe + emotion ∗ Gruppe + T2 Lag ∗ masking ∗ Gruppe	0.006	4.785e -4	0.079	0.002	7.901
T2 Lag + masking + emotion + Gruppe + T2 Lag ∗ emotion + T2 Lag ∗ Gruppe	0.006	4.672e -4	0.078	0.002	4.291
T2 Lag + masking + emotion + Gruppe + masking ∗ emotion + T2 Lag ∗ Gruppe	0.006	3.699e -4	0.061	0.001	5.284
T2 Lag + emotion + T2 Lag ∗ emotion	0.006	3.549e -4	0.059	0.001	3.471
T2 Lag + masking + emotion + Gruppe + T2 Lag ∗ Gruppe + emotion ∗ Gruppe	0.006	3.376e -4	0.056	0.001	4.867
T2 Lag + masking + emotion + Gruppe + T2 Lag ∗ masking + T2 Lag ∗ emotion + masking ∗ emotion + T2 Lag ∗ Gruppe + masking ∗ Gruppe + emotion ∗ Gruppe	0.006	2.767e -4	0.046	9.360e -4	21.067
T2 Lag + masking + emotion + Gruppe + T2 Lag ∗ emotion + masking ∗ emotion + T2 Lag ∗ Gruppe + masking ∗ Gruppe + emotion ∗ Gruppe + masking ∗ emotion ∗ Gruppe	0.006	2.569e -4	0.043	8.693e -4	11.848
T2 Lag + masking + emotion + Gruppe + T2 Lag ∗ masking + T2 Lag ∗ emotion + masking ∗ emotion + T2 Lag ∗ Gruppe + masking ∗ Gruppe + T2 Lag ∗ masking ∗ emotion	0.006	2.495e -4	0.041	8.440e -4	6.780
T2 Lag + masking + emotion + Gruppe + T2 Lag ∗ masking + T2 Lag ∗ emotion + T2 Lag ∗ Gruppe + masking ∗ Gruppe + emotion ∗ Gruppe + T2 Lag ∗ emotion ∗ Gruppe	0.006	2.265e -4	0.038	7.663e -4	5.522
T2 Lag + masking + emotion + Gruppe + T2 Lag ∗ masking + masking ∗ emotion + T2 Lag ∗ Gruppe + masking ∗ Gruppe + emotion ∗ Gruppe + masking ∗ emotion ∗ Gruppe	0.006	2.124e -4	0.035	7.187e -4	5.820
T2 Lag + emotion + Gruppe + emotion ∗ Gruppe	0.006	2.122e -4	0.035	7.178e -4	50.431
T2 Lag + masking + emotion	0.006	2.042e -4	0.034	6.909e -4	4.384
T2 Lag + masking + Gruppe	0.006	2.003e -4	0.033	6.776e -4	8.023
T2 Lag + masking + emotion + Gruppe + T2 Lag ∗ emotion + masking ∗ emotion + T2 Lag ∗ Gruppe + masking ∗ Gruppe + emotion ∗ Gruppe + T2 Lag ∗ emotion ∗ Gruppe	0.006	1.913e -4	0.032	6.474e -4	5.409
T2 Lag + masking + emotion + Gruppe + T2 Lag ∗ masking + T2 Lag ∗ emotion + masking ∗ Gruppe	0.006	1.726e -4	0.029	5.839e -4	11.659
T2 Lag + emotion + Gruppe + T2 Lag ∗ emotion + T2 Lag ∗ Gruppe + emotion ∗ Gruppe + T2 Lag ∗ emotion ∗ Gruppe	0.006	1.520e -4	0.025	5.141e -4	7.171
T2 Lag + masking + emotion + Gruppe + T2 Lag ∗ masking + masking ∗ emotion + masking ∗ Gruppe	0.006	1.272e -4	0.021	4.302e -4	7.030
T2 Lag + masking + emotion + Gruppe + T2 Lag ∗ emotion + masking ∗ emotion + masking ∗ Gruppe	0.006	1.271e -4	0.021	4.300e -4	6.267
T2 Lag + masking + emotion + Gruppe + T2 Lag ∗ emotion + masking ∗ Gruppe + emotion ∗ Gruppe	0.006	1.252e -4	0.021	4.236e -4	8.420
T2 Lag + emotion + Gruppe + T2 Lag ∗ emotion	0.006	1.224e -4	0.020	4.141e -4	4.113
T2 Lag + masking + emotion + Gruppe + T2 Lag ∗ masking + T2 Lag ∗ emotion + masking ∗ emotion + T2 Lag ∗ Gruppe + masking ∗ Gruppe + emotion ∗ Gruppe + T2 Lag ∗ masking ∗ Gruppe + masking ∗ emotion ∗ Gruppe	0.006	1.221e -4	0.020	4.132e -4	8.808
T2 Lag + masking + T2 Lag ∗ masking	0.006	1.153e -4	0.019	3.902e -4	3.347
T2 Lag + masking + emotion + Gruppe + T2 Lag ∗ masking + masking ∗ Gruppe + emotion ∗ Gruppe	0.006	1.066e -4	0.018	3.606e -4	4.575
T2 Lag + masking + emotion + Gruppe + T2 Lag ∗ masking + T2 Lag ∗ emotion + T2 Lag ∗ Gruppe	0.006	1.023e -4	0.017	3.461e -4	5.278
T2 Lag + masking + emotion + Gruppe + T2 Lag ∗ masking + T2 Lag ∗ emotion + masking ∗ emotion + T2 Lag ∗ Gruppe + masking ∗ Gruppe + emotion ∗ Gruppe + T2 Lag ∗ masking ∗ Gruppe + T2 Lag ∗ emotion ∗ Gruppe	0.006	9.900e -5	0.016	3.349e -4	6.838
T2 Lag + masking + emotion + Gruppe + masking ∗ emotion + masking ∗ Gruppe + emotion ∗ Gruppe	0.006	9.690e -5	0.016	3.278e -4	8.339
T2 Lag + masking + emotion + Gruppe + T2 Lag ∗ masking + T2 Lag ∗ emotion + masking ∗ emotion + T2 Lag ∗ Gruppe + masking ∗ Gruppe + emotion ∗ Gruppe + T2 Lag ∗ masking ∗ emotion + T2 Lag ∗ masking ∗ Gruppe	0.006	9.348e -5	0.016	3.163e -4	5.242
T2 Lag + masking + emotion + Gruppe + T2 Lag ∗ masking + masking ∗ emotion + T2 Lag ∗ Gruppe	0.006	9.227e -5	0.015	3.122e -4	8.740
T2 Lag + masking + emotion + Gruppe + T2 Lag ∗ emotion + masking ∗ emotion + T2 Lag ∗ Gruppe	0.006	8.581e -5	0.014	2.903e -4	5.706
T2 Lag + masking + emotion + Gruppe + T2 Lag ∗ emotion + T2 Lag ∗ Gruppe + emotion ∗ Gruppe	0.006	8.268e -5	0.014	2.797e -4	6.339
T2 Lag + masking + emotion + Gruppe + T2 Lag ∗ Gruppe + emotion ∗ Gruppe	0.006	7.215e -5	0.012	2.441e -4	5.069
T2 Lag + masking + emotion + Gruppe + masking ∗ emotion + T2 Lag ∗ Gruppe + emotion ∗ Gruppe	0.006	6.688e -5	0.011	2.263e -4	7.943
T2 Lag + masking + emotion + Gruppe	0.006	6.434e -5	0.011	2.177e -4	4.866
T2 Lag + masking + emotion + T2 Lag ∗ masking	0.006	5.422e -5	0.009	1.834e -4	10.718
T2 Lag + masking + emotion + Gruppe + T2 Lag ∗ emotion + masking ∗ emotion + T2 Lag ∗ Gruppe + masking ∗ Gruppe + emotion ∗ Gruppe + T2 Lag ∗ emotion ∗ Gruppe + masking ∗ emotion ∗ Gruppe	0.006	5.357e -5	0.009	1.812e -4	11.903
T2 Lag + masking + emotion + Gruppe + T2 Lag ∗ masking + T2 Lag ∗ emotion + masking ∗ Gruppe + emotion ∗ Gruppe	0.006	4.575e -5	0.008	1.548e -4	40.683
T2 Lag + masking + emotion + Gruppe + T2 Lag ∗ masking + T2 Lag ∗ emotion + masking ∗ emotion + T2 Lag ∗ Gruppe + masking ∗ Gruppe + emotion ∗ Gruppe + masking ∗ emotion ∗ Gruppe	0.006	4.490e -5	0.007	1.519e -4	5.784
T2 Lag + masking + emotion + T2 Lag ∗ emotion	0.006	4.427e -5	0.007	1.498e -4	3.721
T2 Lag + masking + emotion + Gruppe + T2 Lag ∗ masking + T2 Lag ∗ emotion + masking ∗ emotion + T2 Lag ∗ Gruppe + masking ∗ Gruppe + emotion ∗ Gruppe + T2 Lag ∗ masking ∗ emotion	0.006	4.259e -5	0.007	1.441e -4	6.783
T2 Lag + masking + emotion + Gruppe + T2 Lag ∗ masking + T2 Lag ∗ emotion + masking ∗ emotion + T2 Lag ∗ Gruppe + masking ∗ Gruppe + emotion ∗ Gruppe + T2 Lag ∗ emotion ∗ Gruppe	0.006	4.176e -5	0.007	1.413e -4	9.705

Model Comparison

Models	P(M)	P(M|data)	BF_M	BF_{10}	error %

Gruppe + T2 Lag ∗ emotion ∗ Gruppe

Note. All models include subject

T2 Lag + masking + Gruppe + T2 Lag ∗ masking	0.006	4.070e -5	0.007	1.377e -4	7.058
T2 Lag + masking + emotion + masking ∗ emotion	0.006	3.697e -5	0.006	1.251e -4	7.004
T2 Lag + masking + emotion + Gruppe + T2 Lag ∗ masking + T2 Lag ∗ emotion + masking ∗ emotion + T2 Lag ∗ Gruppe + masking ∗ Gruppe + emotion ∗ Gruppe + T2 Lag ∗ masking ∗ emotion + T2 Lag ∗ masking ∗ Gruppe + masking ∗ emotion ∗ Gruppe	0.006	2.866e -5	0.005	9.698e -5	11.018
T2 Lag + masking + emotion + Gruppe + T2 Lag ∗ masking + T2 Lag ∗ emotion + masking ∗ emotion + masking ∗ Gruppe	0.006	2.761e -5	0.005	9.341e -5	6.763
T2 Lag + masking + emotion + Gruppe + T2 Lag ∗ masking + T2 Lag ∗ emotion + masking ∗ emotion + T2 Lag ∗ Gruppe + masking ∗ Gruppe + emotion ∗ Gruppe + T2 Lag ∗ masking ∗ Gruppe + T2 Lag ∗ emotion ∗ Gruppe + masking ∗ emotion ∗ Gruppe	0.006	2.746e -5	0.005	9.290e -5	8.703
T2 Lag + masking + emotion + Gruppe + masking ∗ emotion + masking ∗ Gruppe + emotion ∗ Gruppe + masking ∗ emotion ∗ Gruppe	0.006	2.467e -5	0.004	8.346e -5	6.465
T2 Lag + masking + emotion + Gruppe + T2 Lag ∗ masking + T2 Lag ∗ emotion + masking ∗ emotion + T2 Lag ∗ Gruppe + masking ∗ Gruppe + emotion ∗ Gruppe + T2 Lag ∗ masking ∗ emotion + T2 Lag ∗ masking ∗ Gruppe + T2 Lag ∗ emotion ∗ Gruppe	0.006	2.415e -5	0.004	8.169e -5	8.145
T2 Lag + masking + emotion + Gruppe + T2 Lag ∗ masking + masking ∗ emotion + masking ∗ Gruppe + emotion ∗ Gruppe	0.006	2.168e -5	0.004	7.334e -5	6.989
T2 Lag + masking + emotion + Gruppe + T2 Lag ∗ emotion + masking ∗ emotion + masking ∗ Gruppe + emotion ∗ Gruppe	0.006	2.153e -5	0.004	7.284e -5	5.996
T2 Lag + emotion + Gruppe + T2 Lag ∗ emotion + emotion ∗ Gruppe	0.006	2.040e -5	0.003	6.902e -5	3.710
T2 Lag + masking + emotion + Gruppe + T2 Lag ∗ masking + T2 Lag ∗ emotion + masking ∗ emotion + T2 Lag ∗ Gruppe + masking ∗ Gruppe + emotion ∗ Gruppe + T2 Lag ∗ masking ∗ emotion + T2 Lag ∗ emotion ∗ Gruppe	0.006	1.943e -5	0.003	6.575e -5	35.853
T2 Lag + masking + emotion + Gruppe + T2 Lag ∗ emotion + T2 Lag ∗ Gruppe + emotion ∗ Gruppe + T2 Lag ∗ emotion ∗ Gruppe	0.006	1.848e -5	0.003	6.251e -5	12.223
T2 Lag + masking + emotion + Gruppe + T2 Lag ∗ masking + T2 Lag ∗ emotion + masking ∗ emotion + T2 Lag ∗ Gruppe	0.006	1.775e -5	0.003	6.004e -5	4.587
T2 Lag + masking + emotion + Gruppe + T2 Lag ∗ masking + T2 Lag ∗ emotion + T2 Lag ∗ Gruppe + emotion ∗ Gruppe	0.006	1.693e -5	0.003	5.729e -5	6.195
T2 Lag + masking + emotion + Gruppe + T2 Lag ∗ masking + masking ∗ emotion + T2 Lag ∗ Gruppe + emotion ∗ Gruppe	0.006	1.490e -5	0.002	5.042e -5	9.043
T2 Lag + masking + emotion + Gruppe + T2 Lag ∗ emotion	0.006	1.447e -5	0.002	4.896e -5	4.809
T2 Lag + masking + emotion + Gruppe + T2 Lag ∗ masking	0.006	1.341e -5	0.002	4.536e -5	3.977
T2 Lag + masking + emotion + Gruppe + T2 Lag ∗ emotion + masking ∗ emotion + T2 Lag ∗ Gruppe + emotion ∗ Gruppe	0.006	1.317e -5	0.002	4.456e -5	4.486
T2 Lag + masking + emotion + Gruppe + emotion ∗ Gruppe	0.006	1.254e -5	0.002	4.242e -5	9.734
T2 Lag + masking + emotion + T2 Lag ∗ emotion + masking ∗ emotion	0.006	1.226e -5	0.002	4.149e -5	25.660
T2 Lag + masking + emotion + Gruppe + T2 Lag ∗ masking + T2 Lag ∗ emotion + masking ∗ emotion + T2 Lag ∗ Gruppe + masking ∗ Gruppe + emotion ∗ Gruppe + T2 Lag ∗ emotion ∗ Gruppe + masking ∗ emotion ∗ Gruppe	0.006	1.219e -5	0.002	4.125e -5	10.083
T2 Lag + masking + emotion + Gruppe + masking ∗ emotion	0.006	1.149e -5	0.002	3.887e -5	4.051
T2 Lag + masking + emotion + T2 Lag ∗ masking + T2 Lag ∗ emotion	0.006	1.145e -5	0.002	3.873e -5	8.724
T2 Lag + masking + emotion + Gruppe + T2 Lag ∗ masking + T2 Lag ∗ emotion + masking ∗ emotion + T2 Lag ∗ Gruppe + masking ∗ Gruppe + emotion ∗ Gruppe + T2 Lag ∗ masking ∗ emotion + masking ∗ emotion ∗ Gruppe	0.006	1.087e -5	0.002	3.677e -5	9.281
T2 Lag + masking + emotion + T2 Lag ∗ masking + masking ∗ emotion	0.006	8.590e -6	0.001	2.906e -5	6.284
T2 Lag + masking + emotion + Gruppe + T2 Lag ∗ masking + T2 Lag ∗ emotion + masking ∗ emotion + masking ∗ Gruppe + T2 Lag ∗ masking ∗ emotion	0.006	7.199e -6	0.001	2.436e -5	8.683
T2 Lag + masking + emotion + Gruppe + T2 Lag ∗ emotion + masking ∗ emotion + masking ∗ Gruppe + emotion ∗ Gruppe + masking ∗ emotion ∗ Gruppe	0.006	6.562e -6	0.001	2.220e -5	16.434
T2 Lag + masking + emotion + Gruppe + T2 Lag ∗ masking + T2 Lag ∗ emotion + masking ∗ emotion + T2 Lag ∗ Gruppe + masking ∗ Gruppe + emotion ∗ Gruppe + T2 Lag ∗ masking ∗ emotion + T2 Lag ∗ masking ∗ Gruppe + T2 Lag ∗ emotion ∗ Gruppe + masking ∗ emotion ∗ Gruppe	0.006	6.151e -6	0.001	2.081e -5	16.522
T2 Lag + masking + emotion + Gruppe + T2 Lag ∗ masking + masking ∗ emotion + masking ∗ Gruppe + emotion ∗ Gruppe + masking ∗ emotion ∗ Gruppe	0.006	5.103e -6	8.470e -4	1.726e -5	5.766
T2 Lag + masking + emotion + Gruppe + T2 Lag ∗ masking + T2 Lag ∗ emotion + masking ∗ emotion + T2 Lag ∗ Gruppe + T2 Lag ∗ masking ∗ emotion	0.006	5.054e -6	8.389e -4	1.710e -5	10.534
T2 Lag + masking + emotion + Gruppe + T2 Lag ∗ masking + T2 Lag ∗ emotion + masking ∗ emotion + masking ∗ Gruppe + emotion ∗ Gruppe	0.006	4.951e -6	8.218e -4	1.675e -5	10.296
T2 Lag + masking + emotion + Gruppe + T2 Lag ∗ emotion + masking ∗ emotion + T2 Lag ∗ Gruppe + emotion ∗ Gruppe + T2 Lag ∗ emotion ∗ Gruppe	0.006	4.072e -6	6.760e -4	1.378e -5	12.063
T2 Lag + masking + emotion + Gruppe + T2 Lag ∗ masking + T2 Lag ∗ emotion + T2 Lag ∗ Gruppe + emotion ∗ Gruppe + T2 Lag ∗ emotion ∗ Gruppe	0.006	3.982e -6	6.610e -4	1.347e -5	5.284
T2 Lag + masking + emotion + Gruppe + T2 Lag ∗ masking + T2 Lag ∗ emotion + masking ∗ emotion + T2 Lag ∗ Gruppe + emotion ∗ Gruppe	0.006	3.835e -6	6.367e -4	1.298e -5	14.358
T2 Lag + masking + emotion + Gruppe + T2 Lag ∗ masking + T2 Lag ∗ emotion	0.006	3.271e -6	5.431e -4	1.107e -5	4.994
T2 Lag + masking + emotion + Gruppe + T2 Lag ∗ emotion + emotion ∗ Gruppe	0.006	3.159e -6	5.243e -4	1.069e -5	21.772
T2 Lag + masking + emotion + Gruppe + T2 Lag ∗ emotion + masking ∗ emotion	0.006	2.905e -6	4.822e -4	9.827e -6	5.767
T2 Lag + masking + emotion + Gruppe + T2 Lag ∗ masking + masking ∗ emotion	0.006	2.577e -6	4.278e -4	8.720e -6	7.476
T2 Lag + masking + emotion + Gruppe + T2 Lag ∗ masking + emotion ∗ Gruppe	0.006	2.570e -6	4.266e -4	8.695e -6	5.792
T2 Lag + masking + emotion + Gruppe + T2 Lag ∗ masking + T2 Lag ∗ emotion + masking ∗ emotion + T2 Lag ∗ Gruppe + masking ∗ Gruppe + emotion ∗ Gruppe + T2 Lag ∗ masking ∗ emotion + T2 Lag ∗ emotion ∗ Gruppe + masking ∗ emotion ∗ Gruppe	0.006	2.330e -6	3.868e -4	7.883e -6	6.271
T2 Lag + masking + emotion + Gruppe + masking ∗ emotion + emotion ∗ Gruppe	0.006	2.001e -6	3.321e -4	6.769e -6	4.402
T2 Lag + masking + emotion + T2 Lag ∗ masking + T2 Lag ∗ emotion + masking ∗ emotion	0.006	1.892e -6	3.141e -4	6.403e -6	8.599
T2 Lag + masking + emotion + Gruppe + T2 Lag ∗ masking + T2 Lag ∗ emotion + masking ∗ emotion + T2 Lag ∗ Gruppe + masking ∗ Gruppe + emotion ∗ Gruppe + T2 Lag ∗ masking ∗ emotion + T2 Lag ∗ masking ∗ Gruppe + T2 Lag ∗ emotion ∗ Gruppe + masking ∗ emotion ∗ Gruppe + T2 Lag ∗ masking ∗ emotion ∗ Gruppe	0.006	1.828e -6	3.034e -4	6.184e -6	6.636
T2 Lag + masking + emotion + Gruppe + T2 Lag ∗ masking + T2 Lag ∗ emotion + masking ∗ emotion + masking ∗ Gruppe + emotion ∗ Gruppe + masking ∗ emotion ∗ Gruppe	0.006	1.248e -6	2.071e -4	4.222e -6	6.260
T2 Lag + masking + emotion + Gruppe + T2 Lag ∗ masking + T2 Lag ∗ emotion + masking ∗ emotion + masking ∗ Gruppe + emotion ∗ Gruppe + T2 Lag ∗ masking ∗ emotion	0.006	9.301e -7	1.544e -4	3.147e -6	5.110
T2 Lag + masking + emotion + Gruppe + T2 Lag ∗ masking + T2 Lag ∗ emotion + masking ∗ emotion + T2 Lag ∗ Gruppe + emotion ∗ Gruppe + T2 Lag ∗ masking ∗ emotion	0.006	7.695e -7	1.277e -4	2.603e -6	10.151
T2 Lag + masking + emotion + Gruppe + T2 Lag ∗ masking + T2 Lag ∗ emotion + masking ∗ emotion + T2 Lag ∗ Gruppe + emotion ∗ Gruppe + T2 Lag ∗ emotion ∗ Gruppe	0.006	7.139e -7	1.185e -4	2.415e -6	5.280
T2 Lag + masking + emotion + Gruppe + T2 Lag ∗ masking + T2 Lag ∗ emotion + emotion ∗ Gruppe	0.006	6.034e -7	1.002e -4	2.041e -6	11.148
T2 Lag + masking + emotion + Gruppe + T2 Lag ∗ masking + T2 Lag ∗ emotion + masking ∗ emotion	0.006	5.619e -7	9.328e -5	1.901e -6	4.786
T2 Lag + masking + emotion + Gruppe + T2 Lag ∗ emotion + masking ∗ emotion + emotion ∗ Gruppe	0.006	4.751e -7	7.886e -5	1.607e -6	4.988
T2 Lag + masking + emotion + Gruppe + T2 Lag ∗ masking + masking ∗ emotion + emotion ∗ Gruppe	0.006	4.213e -7	6.993e -5	1.425e -6	4.472
T2 Lag + masking + emotion + T2 Lag ∗ masking + T2 Lag ∗ emotion + masking ∗ emotion + T2 Lag ∗ masking ∗ emotion	0.006	3.806e -7	6.319e -5	1.288e -6	4.350
T2 Lag + masking + emotion + Gruppe + T2 Lag ∗ masking + T2 Lag ∗ emotion + masking ∗ emotion + masking ∗ Gruppe + emotion ∗ Gruppe + T2 Lag ∗ masking ∗ emotion + masking ∗ emotion ∗ Gruppe	0.006	2.913e -7	4.835e -5	9.854e -7	7.867
T2 Lag + masking + emotion + Gruppe + T2 Lag ∗ masking + T2 Lag ∗ emotion + masking ∗ emotion + T2 Lag ∗ Gruppe + emotion ∗ Gruppe + T2 Lag ∗ masking ∗ emotion + T2 Lag ∗ emotion ∗ Gruppe	0.006	1.563e -7	2.594e -5	5.287e -7	7.387
T2 Lag + masking + emotion + Gruppe + T2 Lag ∗ masking + T2 Lag ∗ emotion + masking ∗ emotion + T2 Lag ∗ masking ∗ emotion	0.006	1.355e -7	2.250e -5	4.586e -7	7.255
T2 Lag + masking + emotion + Gruppe + T2 Lag ∗ masking + T2 Lag ∗ emotion + masking ∗ emotion + emotion ∗ Gruppe	0.006	1.334e -7	2.215e -5	4.514e -7	34.168
T2 Lag + masking + emotion + Gruppe + T2 Lag ∗ masking + T2 Lag ∗ emotion + masking ∗ emotion + emotion ∗ Gruppe + T2 Lag ∗ masking ∗ emotion	0.006	2.859e -8	4.746e -6	9.674e -8	10.081
Null model (incl. subject)	0.006	1.898e -40	3.150e -38	6.420e -40	2.910
Gruppe	0.006	4.720e -41	7.836e -39	1.597e -40	2.966
emotion	0.006	4.043e -41	6.711e -39	1.368e -40	3.052
masking	0.006	2.316e -41	3.845e -39	7.836e -41	3.219
masking + Gruppe + masking ∗ Gruppe	0.006	1.847e -41	3.067e -39	6.251e -41	3.652
emotion + Gruppe	0.006	1.006e -41	1.670e -39	3.403e -41	3.146
masking + Gruppe	0.006	6.120e -42	1.016e -39	2.071e -41	4.270
masking + emotion	0.006	4.895e -42	8.126e -40	1.656e -41	3.229
masking + emotion + Gruppe + masking ∗ Gruppe	0.006	4.136e -42	6.866e -40	1.399e -41	5.356
emotion + Gruppe + emotion ∗ Gruppe	0.006	1.760e -42	2.922e -40	5.955e -42	3.894
masking + emotion + Gruppe	0.006	1.226e -42	2.035e -40	4.147e -42	3.393
masking + emotion + masking ∗ emotion	0.006	9.096e -43	1.510e -40	3.077e -42	4.853
masking + emotion + Gruppe + masking ∗ emotion + masking ∗ Gruppe	0.006	7.213e -43	1.197e -40	2.441e -42	9.834
masking + emotion + Gruppe + masking ∗ Gruppe + emotion ∗ Gruppe	0.006	7.066e -43	1.173e -40	2.391e -42	8.656
masking + emotion + Gruppe + emotion ∗ Gruppe	0.006	2.355e -43	3.909e -41	7.967e -43	4.911
masking + emotion + Gruppe + masking ∗ emotion	0.006	2.179e -43	3.617e -41	7.372e -43	4.194
masking + emotion + Gruppe + masking ∗ emotion + masking ∗ Gruppe + emotion ∗ Gruppe	0.006	1.169e -43	1.941e -41	3.956e -43	4.717
masking + emotion + Gruppe + masking ∗ emotion + emotion ∗ Gruppe	0.006	3.890e -44	6.458e -42	1.316e -43	5.732
masking + emotion + Gruppe + masking ∗ emotion + masking ∗ Gruppe + emotion ∗ Gruppe + masking ∗ emotion ∗ Gruppe	0.006	3.082e -44	5.117e -42	1.043e -43	8.181

Note. All models include subject

Analysis of Effects

Effects	P(incl)	P(incl|data)	BF_{incl}
T2 Lag	0.114	0.023	6.535e +37
masking	0.114	0.009	0.124
emotion	0.114	0.237	0.397
Gruppe	0.114	0.003	0.395
T2 Lag ∗ masking	0.299	0.121	0.233
T2 Lag ∗ emotion	0.299	0.073	0.224
masking ∗ emotion	0.299	0.060	0.191
Gruppe ∗ T2 Lag	0.299	0.686	33.517
Gruppe ∗ masking	0.299	0.626	54.863
Gruppe ∗ emotion	0.299	0.058	0.171
T2 Lag ∗ masking ∗ emotion	0.114	0.001	0.209
Gruppe ∗ T2 Lag ∗ masking	0.114	0.284	2.460
Gruppe ∗ T2 Lag ∗ emotion	0.114	0.003	0.253
Gruppe ∗ masking ∗ emotion	0.114	0.002	0.241
Gruppe ∗ T2 Lag ∗ masking ∗ emotion	0.006	1.828e -6	0.297

Note. Compares models that contain the effect to equivalent models stripped of the effect. Higher-order interactions are excluded. Analysis suggested by Sebastiaan Mathôt.
