# Supplementary material for: No Evidence for an Awareness-Dependent Emotional Modulation of the Attentional Blink
Source: Front Psychol. 2019 Oct 25;10:2422. doi: 10.3389/fpsyg.2019.02422 (PMC6842977; doi:10.3389/fpsyg.2019.02422)
Supplement: Supplementary file 4 [file Data_Sheet_1.DOCX]

Determination of sample size

Sample size was determined based on the details provided by Qian et al. (2012). Qian et al. report the results of an analysis of their pooled data, i.e., including masked and unmasked emotional and neutral faces. They find an interaction between emotional condition (within group factor) and awareness level (between group factor), with a partial eta square of 0.406 (f=.823, large effect size). In the present study, both factors were within group factors. According to calculations done with G*Power 3.1 (Faul, F., Erdfelder, E., Lang, A.-G., & Buchner, A. (2007). G*Power 3: A flexible statistical power analysis program for the social, behavioral, and biomedical sciences. Behavior Research Methods, 39, 175-191), to detect an interaction effect of similar size in a within-group design with an alpha error probability of .05 and a Power of 0.8, a sample size of N=6 would be sufficient. We opted to base our calculations on a more conservative assumption of modest effect size of f = 0.4, corresponding to roughly half the effect size reported by Qian et al. According to our G*Power calculations, for an effect size of f=.4 a sample size of N=15 is sufficient. We collect data from N=20 participants in our experiments to have a safety margin in case participants had to be excluded from analysis. As this was not the case all 20 data sets could be used, resulting in an actual Power of 0.92 to detect an effect of size f=.4 with an alpha error probability of p=.05.
